# Plant-derived SAC domain of PAR-4 (Prostate Apoptosis Response 4) exhibits growth inhibitory effects in prostate cancer cells

**DOI:** 10.3389/fpls.2015.00822

**Published:** 2015-10-07

**Authors:** Shayan Sarkar, Sumeet Jain, Vineeta Rai, Dipak K. Sahoo, Sumita Raha, Sujit Suklabaidya, Shantibhusan Senapati, Vivek M. Rangnekar, Indu B. Maiti, Nrisingha Dey

**Affiliations:** ^1^Department of Gene Function and Regulation, Institute of Life Sciences, Department of Biotechnology, Government of IndiaBhubaneswar, India; ^2^Department of Translational Research and Technology Development, Institute of Life Sciences, Department of Biotechnology, Government of IndiaBhubaneswar, India; ^3^Manipal UniversityManipal, India; ^4^Kentucky Tobacco Research & Development Center, Plant Genetic Engineering Research and Services, College of Agriculture, Food and Environment, University of Kentucky, LexingtonKY, USA; ^5^Department of Agronomy, Iowa State University, AmesIA, USA; ^6^Department of Radiation Oncology, Feinberg School of Medicine, Northwestern University, ChicagoIL, USA; ^7^Department of Radiation Medicine, Markey Cancer Center, University of Kentucky, LexingtonKY, USA

**Keywords:** fusion protein, SAC domain of Par-4, transgenic plant, glycosylation, molecular farming, apoptosis

## Abstract

The gene *Par-4* (Prostate Apoptosis Response 4) was originally identified in prostate cancer cells undergoing apoptosis and its product Par-4 showed cancer specific pro-apoptotic activity. Particularly, the SAC domain of Par-4 (SAC-Par-4) selectively kills cancer cells leaving normal cells unaffected. The therapeutic significance of bioactive SAC-Par-4 is enormous in cancer biology; however, its large scale production is still a matter of concern. Here we report the production of SAC-Par-4-GFP fusion protein coupled to translational enhancer sequence (5′ AMV) and apoplast signal peptide (aTP) in transgenic *Nicotiana tabacum* cv. Samsun NN plants under the control of a unique recombinant promoter M24. Transgene integration was confirmed by genomic DNA PCR, Southern and Northern blotting, Real-time PCR, and Nuclear run-on assays. Results of Western blot analysis and ELISA confirmed expression of recombinant SAC-Par-4-GFP protein and it was as high as 0.15% of total soluble protein. In addition, we found that targeting of plant recombinant SAC-Par-4-GFP to the apoplast and endoplasmic reticulum (ER) was essential for the stability of plant recombinant protein in comparison to the bacterial derived SAC-Par-4. Deglycosylation analysis demonstrated that ER-targeted SAC-Par-4-GFP-SEKDEL undergoes O-linked glycosylation unlike apoplast-targeted SAC-Par-4-GFP. Furthermore, various *in vitro* studies like mammalian cells proliferation assay (MTT), apoptosis induction assays, and NF-κB suppression suggested the cytotoxic and apoptotic properties of plant-derived SAC-Par-4-GFP against multiple prostate cancer cell lines. Additionally, pre-treatment of MAT-LyLu prostate cancer cells with purified SAC-Par-4-GFP significantly delayed the onset of tumor in a syngeneic rat prostate cancer model. Taken altogether, we proclaim that plant made SAC-Par-4 may become a useful alternate therapy for effectively alleviating cancer in the new era.

## Introduction

Prostate cancer is the second most common cause of cancer and the sixth leading cause of cancer death among men worldwide (Ferlay et al., 2010; Siegel et al., 2014). Prostate cancer is associated with the inability of prostatic epithelial cells to undergo apoptosis rather than with enhanced cell proliferation. Spontaneous metastasis of tumors from the primary site to distant tissues causes mortality in advanced cancer patients (Cooperberg et al., 2009).

Prostate apoptosis response-4 (Par-4) protein (340 amino acids) is capable of promoting apoptosis in cancer cells and causes regression of tumors in animal models (Gurumurthy and Rangnekar, 2004). The Par-4 protein is ubiquitously expressed in numerous tissues among various species, encoded by pro-apoptotic *Par-4* gene located on human chromosome 12q21, rat chromosome 7q21, and mouse chromosome 10D1 (Johnstone et al., 1996; El-Guendy and Rangnekar, 2003). It is a multi-domain protein that is structurally segmented into leucine-zipper domain (LZ) at the carboxyl terminal region, two nuclear localization sequences (NLS1, NLS2), a nuclear export sequence (El-Guendy et al., 2003) and a unique SAC (Selective for Apoptosis of Cancer Cells) domain including the NLS2 domain (Hebbar et al., 2012). Sells et al. (1994) identified *Par-4* as an immediate early apoptotic gene through differential hybridization screening of rat AT-3 androgen dependent prostate cancer cell line exposed to ionomycin for the induction of apoptosis. Consistent with its pro-apoptotic function, Par-4 is found to be frequently deleted in pancreatic and gastric cancer (Kimura and Gelmann, 2000; Boehrer et al., 2001), down-regulated in renal-cell carcinomas (Cook et al., 1999), neuroblastoma (Kogel et al., 2001), acute lymphoblastic, leukemia, chronic lymphocytic leukemia (Boehrer et al., 2001), endometrial cancer (Moreno-Bueno et al., 2007), and silenced in endometrial cancer cell lines SKUT1B and AN3CA (Moreno-Bueno et al., 2007).

Interestingly, a 59 amino acid long SAC domain (amino acid coordinates 137–195 in rat Par-4; and 145–204 in human Par-4, respectively) of Par-4 is effective in inducing apoptosis in cancer cells. This domain is 100% conserved in human, rat, and mouse homologs (El-Guendy and Rangnekar, 2003). The SAC domain of Par-4 is the main functional unit for the induction of apoptosis in cancer cells (El-Guendy et al., 2003) and its activity depends on its nuclear entry and phosphorylation at Threonine 155 (Zhao and Rangnekar, 2008). Whole Par-4 and its SAC domain (SAC-Par-4) both can induce apoptosis through intrinsic and extrinsic pathways (Burikhanov et al., 2009; Hebbar et al., 2012). Overexpression of Par-4 or SAC domain induces apoptosis in different cancer cell lines but does not kill normal cells in cell culture studies (Burikhanov et al., 2009). In animal model, systemic overexpression of Par-4 is shown to inhibit tumor growth and metastasis (Zhao et al., 2011). A previous study have shown that the full-length Par-4 interacts with Akt1 (cell survival kinase) through LZ domain to confer cancer cells resistant to apoptosis; however, SAC-domain lacking LZ domain could escape binding to Akt1 and can potentially kill cancer cells (Goswami et al., 2005). The ability of SAC-domain to induce apoptosis in diverse cancer cells can be exploited as potential anti-cancer regimen to induce tumor suppression via apoptosis. Thus SAC-Par-4 is gaining world-wide attention as an effective anti-cancer therapeutics; implying necessities for high-scale production of biologically active SAC-Par-4 protein.

Molecular farming of essential therapeutics/drug molecules in plants have several advantages over their conventional production in bacteria, yeast, or cultured animal or human cell lines (Goldstein and Thomas, 2004; Ko et al., 2005; Ma et al., 2005a,b,c; Daniell, 2006; Fox, 2006; Gleba et al., 2007). These studies clearly demonstrated that transgenic plants could become an effective expression system for profitable production of plant-made products (PMP; Goddijn and Pen, 1995; Daniell et al., 2001). Besides, plant ensures hygienic pharmaceutical production avoiding harmful or lethal contaminants like viruses, toxins, prions, oncogenes (Boehm, 2007).

In the present study, we reported the expression of SAC-Par-4-GFP in transgenic tobacco plants under the control of modified full-length transcript promoter (M24) of the *Mirabilis mosaic virus*. Furthermore, the SAC-Par-4-GFP was fused with translational enhancer sequence (5′ AMV), apoplast targeting sequence (aTP) to increase the transgene stability in plant and to direct the protein into the secretory pathway and, finally, into the apoplast. The stability and functionality of plant-derived SAC-Par-4-GFP was confirmed by different molecular analysis. Alongside, retention to the endoplasmic reticulum (ER) was achieved by adding an ER retrieval signal (SEKDEL) to the C-terminus of the apoplast-targeted SAC-Par-4-GFP construct and transiently expressed in tobacco plant to obtain a glycosylated and proteolytically stable protein. The proteolytic stability of transgenic plant-derived recombinant SAC-Par-4-GFP and transiently expressed SAC-Par-4-GFP-SEKDEL was compared with bacterial SAC-Par-4. Cancer-specific effectiveness and bioactivity of plant-made SAC-Par-4-GFP was confirmed by mammalian cell proliferation assays in PC3, MAT-LyLu, LNCaP prostate cancer cells lines, and HEK293 non-cancerous cells along with apoptosis induction in PC3 and NF-κB suppression activities in PC3, MAT-LyLu, and HEK293 cells. Moreover, the onset of tumor by MAT-LyLu cells pre-treated and co-injected with SAC-Par-4-GFP was studied in Copenhagen rats. Our present findings lay a foundation regarding the anti-prostate cancer activity of plant-derived SAC-Par-4 and hence SAC-Par-4 could become an effective plant-made biologics in controlling prostate cancer. This is a nascent report demonstrating the inhibitory properties of plant-derived SAC-Par-4-GFP on prostate cancer cells’ growth.

## Materials and Methods

### Construction of Chimeric Gene Constructs for Plant Transformation

**FIGURE 1:**
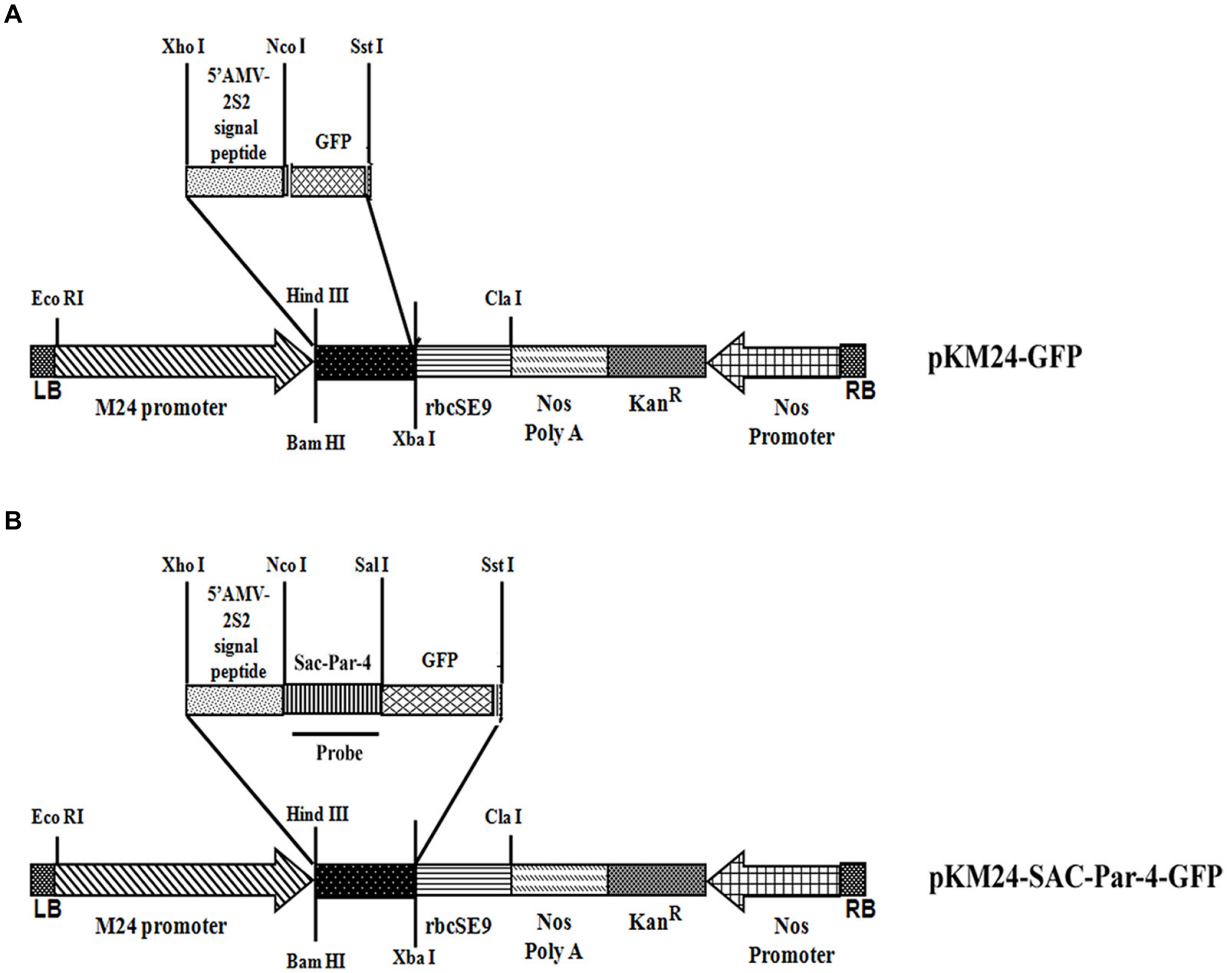
**Schematic representation of the constructs. (A)** Plant expression vector containing *GFP* (pKM24-GFP; VC); **(B)** the synthetic *SAC-Par-4* gene fused in-frame with GFP (pKM24-SAC-Par-4-GFP). LB: left T-DNA border; RB: right T-DNA border; 5′ AMV: a translational enhancer sequence; aTP: the apoplast targeting sequence of the *Arabidopsis* 2S2 protein; M24: recombinant full-length *Mirabilis mosaic virus* promoter, Kan^R^: *neomycin phosphotransferase* II marker gene; rbcSE9: the 3′-terminator sequences (terminators) of the ribulose bisphosphate carboxylase small subunit and Nos PolyA: *nopaline synthase* genes are shown. The *EcoR*I, *Xho*I, *Nco*I, *Sal*I, *Sst*I, *Hind*III, *Bam*HI, *Xba*I, and *Cla*I restriction sites used to assemble these expression vectors are shown. The Southern and Northern hybridization probe (SAC-Par-4) is indicated by thick line in **(B)**.

The rat *Par-4*-SAC domain (GenBank accession no U05989) was fused with GFP to generate SAC-Par-4-GFP recombinant DNA fragment. This sequence was then codon-optimized for *Nicotiana tabacum*. A 35-nucleotide long 5′-untranslated region of AlMV RNA 4 (5′ AMV; translational enhancer) and apoplast targeting sequence (aTP) of *Arabidopsis* 2S2 protein gene (Kroumova et al., 2013) were fused with the normalized coding sequence of SAC-Par-4-GFP. The recombinant sequence thus generated was synthesized by GeneArt (Invitrogen, Life technologies, USA^1^) and cloned into *Xho*I and *Sst*I sites of the binary vector pKM24KH (GenBank accession HM036220) carrying modified full-length transcript promoter (M24) of the *Mirabilis mosaic virus* (Dey and Maiti, 1999a,b) to generate the plasmid pKM24-SAC-Par-4-GFP. The pKM24-GFP construct was also generated and used as vector control. The physical map of different structural components and respective restriction sites of different constructs are represented in **Figure 1**.

### Development of Transgenic Tobacco Plants

Ten independent T_0_ transgenic plant lines expressing pKM24-GFP and pKM24-SAC Par-4-GFP were raised following protocol described earlier (Kumar et al., 2011; Sahoo et al., 2014b; Patro et al., 2015). Subsequently, segregation analysis for T_1_ seeds from each independent line was performed following earlier protocol (Patro et al., 2015). Seeds from T_1_ plants showing appropriate segregation ratios (Kan^R^:Kan^S^= 3:1) were selected and respective T_2_ transgenic plants were raised and maintained in green-house condition.

### Green Fluorescent Protein (GFP) Assay

Total protein from leaves of 8-week-old transgenic pKM24-SAC-Par-4-GFP (L1–L10) and pKM24-GFP (control plant) was extracted following earlier protocol (Verwoerd et al., 1995; Kroumova et al., 2007) and quantified using Bradford (1976) method where BSA served as standard.

Fluorometric quantification of GFP was done following the earlier described protocol (Remans et al., 1999; Sahoo et al., 2014a). The GFP concentration (expressed in μg per mg protein) was measured in total leaf protein extracts from transgenic and vector control plants with the Turner Biosystems Luminometer employing the GFP-UV module using rGFP-S65T (Clontech) as standard. The results were expressed as means ± standard deviation of data from five different samples of the same line (three readings were taken per experiment).

### GFP Visualization by Confocal Laser Scanning Microscopy

The fluorescence images of transgenic tobacco line L3 expressing SAC-Par-4-GFP were captured with Confocal Laser Scanning Microscope (TCS SP5; Leica Microsystems CMS GmbH, D-68165, Mannheim, Germany) using LAS AF (Leica Application Suite Advanced Fluorescence) 1.8.1 build 1390 software as described earlier by Kroumova et al. (2013). GFP expressed in transgenic plant was excited with an argon laser (30%) with AOTF for 488 nm (at 40%; Sahoo et al., 2009), and the fluorescence emissions were collected between 501 and 580 nm with the photomultiplier tube (PMT) detector gain set at 1150 V.

### Integration Assays for *GFP, SAC-Par-4, npt*II, and *rbcSE*9

Genomic DNA (gDNA) from 3-week old T_2_–seedlings of 10 independent lines expressing pKM24-GFP and pKM24-SAC-Par-4-GFP individually were extracted following protocol of Allen et al. (2006). Integration assay for *GFP* gene was performed by PCR amplification of gDNA obtained from each line individually using GFP primer sets (**Table 1**). Likewise, integration of *SAC-Par-4, npt*II, and *rbcSE*9 genes were performed in selected transgenic lines L2, L3, L5, and L6 expressing pKM24-SAC-Par-4-GFP along with pKM24-GFP (VC) in presence of respective gene specific primer sets (**Table 1**). The PCR amplifications of the above-mentioned genes were carried out following the standardized protocol (Kumar et al., 2011).

**Table 1:** List of Primers used.

Primer name	Primer sequence (5′–3′)
SAC-Par-4-GFP-*Xho*I Fp	GCGGGCCTCGAGCGGATCTTTTTA
SAC-Par-4-GFP-*Sst*I Rp	ATGCAGGAGCTCTCACTTGTAAAGCTCATCCAT
SAC-Par-4-GFP-SEKDEL-*Sst*I Rp	ATGCAGGAGCTCTCAAAGCTCATCCTTCTCGGACTT GTAAAGCTCATCCAT
SAC-Par-4-GFP RT Fp	ATGGGTAGAAAGGGAAAGGG
SAC-Par-4-GFP RT Rp	AGCCTCATTCTGAATAGTG
SAC-Par-4 probe FP	ATGGGTAGAAAGGGAAAGGG
SAC-Par-4 probe RP	AGCCTCATTCTGAAT
GFP Fp	GATGTTAACGGACACAAGTTCT
GFP Rp	CATCATCCTTGAAGAAAATAGTCC
Kan Fp	ATGGCAATTACCTTATCCGCAACT
Kan Rp	CGCCTTCTTGACGAGTTCTTCTGA
rbcSE9 Fp	GCGTCCGGATCCGCTTTCGTTCGTATCATCGGTTTC
rbcSE9 Rp	ATGTAGTCTAGATGATGCATGTTGTCAATCAATTGG
Tubulin Fp	ATGAGAGAGTGCATATCGATT
Tubulin Rp	TTCACTGAAGAAGGTGTTGAA

### Southern Blotting

Southern hybridization was carried out according to Sambrook et al. (1989). Briefly, 10 μg of gDNA isolated from four transgenic (L2, L3, L5, and L6 of SAC-Par-4-GFP) and vector control lines were digested overnight with *Xho*I (Fermentas) at 37°C, subsequently electrophoresed on 0.8% TAE-agarose and transferred to a nylon membrane (Hybond-N+, Amersham). Blotted DNA fragments were hybridized with PCR-amplified P^32^-labeled *SAC-Par-4* probe using primers listed in **Table 1**. Following hybridization at 65°C for 16 h, the membrane was washed and subjected to autoradiography.

### Transcript Analysis

Total RNA was extracted from 3-week old transgenic tobacco seedlings expressing pKM24-SAC-Par-4-GFP (L2, L3, L5, and L6) and vector control line pKM24-GFP using Spectrum Plant Total RNA kit (Sigma) following the manufacturer’s instructions. Total RNA was subsequently treated with *DNase*I (Sigma) to obtain DNA free RNA.

Approximately, 10 μg of the above extracted RNA was subjected to Northern blot analysis as described earlier (Ranjan et al., 2012). Briefly, total RNA was electrophoresed on 1.2% (w/v) formaldehyde-agarose gel, blotted onto Hybond-N+ membrane (Amersham), subsequently hybridized with pre-denatured radiolabeled PCR-amplified P^32^-*SAC-Par-4* probe for 16 h at 60°C, washed and autoradiographed.

The quantitative accumulation of *SAC-Par-4-GFP* transcripts in the above transgenic lines was performed using Real-time PCR following protocol reported earlier (Ranjan et al., 2012). Quantitative Real-time PCR was carried out using 5X HOT FIRE pol Eva Green qPCR Mix plus (ROX) employing Real-time PCR machine (MJ Research, Bio-Rad; Model; CFD-3220). The *SAC-Par-4* mRNA levels in different transgenic plants were expressed as fold excess in comparison to the mRNA level of *tubulin*. The fold difference in the transcript levels of *SAC-Par-4* in comparison to that of *tubulin* in different transgenic plants was presented as the mean of three independent biological replicates with respective standard deviation (SD).

### Nuclei Isolation and Nuclear Run-on Assay

Nuclei from 4-week-old tobacco leaves (6 g) of different SAC-Par-4-GFP transgenic lines namely L2, L3, and L5 and wild tobacco plant were isolated and nuclear run-on assay was performed following the protocol described earlier (Folta and Kaufman, 2006). Briefly, 60 μg of the isolated nuclei from individual plants was incubated in nuclear transcription buffer containing 100 μCi of [α- ^32^P] UTP (6,000 Ci m/mol), 37.5 units of RNasin (Promega) and incubated at 30°C for 30 min. Subsequently, the mixture was extracted by phenol: chloroform: isoamyl alcohol (25:24:1), purified and used as a probe for hybridization (approximately 2 × 10^6^ cpm). PCR amplifications of *SAC-Par-4* gene, *npt*II gene, *18S* rRNA (positive control) were carried out using specific set of primers (**Table 1**). Approximately, 500 ng of PCR products along with pBSK plasmid (negative control) were bound with Hybond-N+ nylon membrane (GE Health Care Amersham Hybond^TM^- XL) using dot-blot apparatus. Membrane was cut into four different strips and each strip was hybridized with heat denatured radiolabel transcripts obtained from each lines separately (SAC-Par-4-GFP lines L2, L3, L5, and wild tobacco plant) for 24 h at 65°C. Membranes were washed and autoradiographed.

### Enzyme Linked Immunosorbent Assay (ELISA) and Western Blot Analysis of Transgenic Plants

Total proteins from 6-week-old transgenic tobacco plant under lines L2, L3, L5, L6, and VC were extracted as described above (under GFP assay section). Approximately an aliquot of 5 μg of respective proteins were used to perform Enzyme linked immunosorbent assay (ELISA) following protocol described by Vazquez et al. (1996). The relative accumulation of SAC-Par-4 protein in transgenic lines was estimated according to Patro et al. (2015). Briefly, total protein samples obtained from transgenic seedlings expressing SAC-Par-4 and VC were coated on a 96-well microtiter plate and incubated with primary antibody specific to PAR-4 (R-334, Santa Cruz) for 2 h. Subsequently after two rounds of washing, horse-radish peroxidase conjugated secondary antibody was added. After incubation for 1 h, the plate was washed twice followed by addition of ortho-phenylenediamine substrate. The color development was measured at 492 nm in a microplate reader (Bio-Rad 3550) and converted as a percentage of total extracted protein by reference to an ELISA standard curve constructed with the bacterial purified SAC-Par-4 (described below).

Aliquots of 10 μg of total plant protein extracts obtained from L2, L3, L5, L6, and VC lines (as described earlier) were resolved on 12% SDS-PAGE, and electrophoretically transferred onto a 0.2-μm polyvinylidene difluoride (PVDF) membrane for Western blot analysis. The primary and secondary antibodies used are discussed above. The blot was visualized by ECL chemiluminescence (GE healthcare, UK). β-actin was used as a loading control.

### Construction of Chimeric Gene Construct for Transient Assay

A plant expression vector, pKM24-SAC-Par-4-GFP-SEKDEL was constructed that targeted SAC-Par-4-GFP to the ER. Retention to the ER was achieved by adding an ER retrieval signal (SEKDEL) to the C-terminus of the apoplast-targeted SAC-Par-4-GFP construct. Description of the construct is similar to that pKM24-SAC-Par-4-GFP given in the “Materials and Methods” section. The physical map of the above construct is given in Supplementary Figure S1.

### Agrobacterium-mediated Transient Expression Assays

*Agrobacterium tumefaciens* strain C58C1: pGV3850 was transformed with pKM24-SAC-Par-4-GFP-SEKDEL construct using freeze-thaw method. Agrobacterium harboring the above construct was used to infiltrate the leaves of 10–14-week-old *N. tabacum* following the protocol described earlier (Kapila et al., 1997; Yang et al., 2000). Seventy two hours post-infiltration, the agro-infiltrated tobacco leaves were processed for protein extraction. The concentration of SAC-Par-4-GFP-SEKDEL was quantified using a sandwich ELISA as described before. To confirm the integrity of transiently expressed SAC-Par-4-GFP-SEKDEL, Western blot analysis was performed on the protein extracts with anti-Par-4 antibody.

### Deglycosylation Assay

Protein extracts obtained from transgenic plant (line L3) expressing SAC-Par-4-GFP and transiently expressed SAC-Par-4-GFP-SEKDEL were digested with the deglycosylating enzyme PNGase F, EndoH, and *O*-glycosidase (New England Biolabs; NEB) for 3 h at 37°C, according to the manufacturer’s instructions. Control samples were treated the same, except that no enzyme was added. To check the O-linked glycosylation status of SAC-Par-4-GFP and SAC-Par-4-GFP-SEKDEL, the plant extract was treated with Neuraminidase (NEB), and the proteins were denatured prior to deglycosylation treatment with O-glycosidase. All treated samples were then subjected to Western blot analysis using the anti-PAR-4 antibody.

### Bacterial Purification of SAC-Par-4

The 59 amino acid (aa 137-195) long rat *SAC-Par-4* was cloned in pET-29b vector (Novagen) and expressed in *BL21* (DE3); purified through HisPur^TM^ Cobalt resin (Thermo Scientific) as described earlier (Sahoo et al., 2014a). The purified SAC-Par-4 was dialyzed and concentrated with a centrifugal filter device (Amicon 10 KDa cut-off), and quantified by Bradford (Sigma). Fractions were collected and analyzed on 18% SDS-PAGE.

### Proteolysis Assays by Trypsin

The protection assays of plant-derived SAC-Par-4-GFP (from transgenic line L3), SAC-Par-4-GFP-SEKDEL (transiently expressed) and SAC-Par-4 (bacterial purified) against trypsin digestion was performed according to Tremblay et al. (2011) and Wang et al. (2008). Aliquots of 10 μL protein extracts were removed at various time intervals and boiled for 10 min in 1x SDS-sample buffer, resolved on a 12% SDS-PAGE gel and subjected to Western blot analysis with the anti-PAR-4 antibody in the above assay.

### Partial Purification of Plant-derived SAC-Par-4

Approximately, 50 g of leaves obtained from transgenic tobacco plant line L3 expressing SAC-Par-4-GFP were powdered using liquid nitrogen and homogenized in 150 ml of 1x PBS buffer supplemented by 5 mM EDTA, 1 mM PMSF and 1.5% PVP-40 and subjected to 20% ammonium sulfate cut. The supernatant obtained was dialyzed against 50 mM Tris-HCl (pH 8.0) buffer for 24 h at 4°C and adjusted to 40% ammonium sulfate saturation; centrifuged at 30,000 × *g* for 40 min. The enriched SAC-Par-4-GFP fraction was obtained as pellet. The pellet was solubilized and dialyzed twice against water and once against antibody affinity column buffer consisting of 20 mM Tris-HCl pH 7.0 and 250 mM NaCl at 4°C. Enriched SAC-Par-4-GFP was further purified according to the protocol of Downing et al. (2006). The insoluble materials were removed by centrifugation at 30,000 × *g* for 20 min at 4°C and the supernatant was loaded onto Affi-Gel antibody affinity column (Bio-Rad) in a cold room. The polyclonal anti- Par-4 antibody (R-334; Santa Cruz) was combined with an equal volume of Affi-Gel 15 (Bio-Rad) and mixed at 4°C for 2 h according to the manufacturer’s instructions. Protein was eluted in antibody elution buffer (50 mM sodium citrate pH 4.0, 2 M NaCl) and extensively dialyzed in 1x PBS and concentrated with a centrifugal filter device (Amicon 10 KDa cut-off).

### Cell Culture and Cell Lines

Hormone-independent PC3 (human) and MAT-LyLu (Rat) prostate cancer cell lines were cultured in RPMI (Pan Biotech) and DMEM (Pan Biotech) media, respectively. Hormone- dependent LNCaP (human) cells were maintained in RPMI medium. Non-cancerous HEK293 (human embryonic kidney cells) was cultured in DMEM. All the media were supplemented with 10% heat-inactivated fetal bovine serum, 100 U/ml penicillin (Sigma) and 100 μg/ml streptomycin (Sigma). The cells were incubated at 37°C in a humidified atmosphere of 95% air and 5% CO_2_.

### Cell Viability Assay

Cell viability was determined by the colorimetric MTT (3-(4,5-dimethylthiazol-2-yl)-2,5- diphenyltetrazolium bromide) assay according to Acharya et al. (2014). Briefly, 2 × 10^3^ cells (PC3, MAT-LyLu, LNCaP, and HEK293) were treated with purified SAC-Par-4-GFP and VC protein in different concentrations for 48 h. Subsequently MTT was added, incubated in dark for 30 min and the absorbance was measured at 570 nm using an ELISA reader (Synergy HT, BioTek Instruments Inc., Winooski, VT, USA). The cell-viability was expressed as percentage of control cells and average cell-viability was presented as a mean of three independent experiments with respective standard deviation. All the experiments were performed in triplicate.

### AnnexinV-FITC and PI Staining

PC3 and HEK293 cells were treated with SAC-Par-4-GFP (50 ug/ml) and VC (60 ug/ml) protein separately for 48 h. After treatment, annexinV-FITC and PI staining was performed as per the protocol described in the kit (Alexa Fluor^®^ 488 annexin V/Dead Cell Apoptosis Kit, Invitrogen). Stained cells were analyzed on BD LSRFortessa^TM^ cell analyzer and the resulting fluorescence was taken by FLH-1 channel for green fluorescence and FLH-2 channel for red fluorescence. AnnexinV-FITC-positive and PI-negative cells were considered as early apoptotic cells (Q2) while those positive for both annexinV-FITC and PI were considered as late apoptotic cells (Q4).

### Cell Cycle Analysis

SAC-Par-4-GFP and VC protein treated cells (mentioned above) were processed as per the protocol described in the kit (Tali^®^ Cell Cycle Kit, Invitrogen). The cell cycle analysis was performed on the Tali^®^ Image-Based Cytometer and results were reported as percentage of sub-G_0_ (sub-genomic DNA), G_0_/G_1_, S and G_2_/M cells. The percentage of total cells in Sub-G_0_ fraction was considered as apoptotic fraction.

### Transduction of Luciferase Lentivirus and NF-κB Reporter Assay

Stable NF-κB luciferase reporter cells (PC3-NF-κB-luc, MAT-LyLu-NF-κB-luc and HEK293- NF-κB-luc) were generated using ready-to-transduce replication incompetent lentiviral particles as suggested by Cignal Lenti NFκB Reporter (luc) Kit (Qiagen). Cells were transduced with lentiviral particles at the multiplicity of infection (MOI) 50 and puromycin (1 μg/ml for PC3, HEK293, and 8 μg/ml for MAT-LyLu) was used for selection of stably transduced cells (Jain et al., 2015). The aforementioned lentiviral particle related works were done with the prior permission of Institutional Biosafety Committee, Institute of Life Sciences, Bhubaneswar.

For luciferase assay, 1 × 10^3^ cells were seeded in 96- well plate, grown for 24 h and treated with non-cytotoxic concentration of SAC-Par-4-GFP in the presence of TNF-α (10 ng/ml) for 6 h. Cells were lysed and luciferase expression was estimated using reporter assay kit (Promega, USA). The activity was normalized with protein concentration from each sample.

### Detection of Cleaved Caspase-3 and Cleaved PARP by Western Blotting

PC3 cells were treated with SAC-Par-4-GFP at different concentrations for 48 h and Western blot was performed as described earlier (Plante et al., 2013). Briefly, 40 μg of protein from each sample were resolved on 10% SDS-PAGE, electrophoretically transferred onto a PVDF membrane and incubated with specific anti-caspase-3 (Cell Signaling) and anti-PARP antibody (Cell Signaling). Anti-rabbit-HRP-conjugated antibody (Santa Cruz) was used as secondary antibody and visualized by ECL chemiluminescence (GE healthcare, UK). β-actin was used as a loading control.

### *In Vivo* Experiment

Animal experiments done in our study were conducted as per the animal ethics guidelines and were approved by animal ethics committee of the Institute of Life Sciences (ILS), Bhubaneswar. As we have used MAT-LyLu prostate cancer cell for our *in vitro* study, therefore, we adopt MAT-LyLu rat syngeneic prostate cancer model for our *in vivo* validation (Chang et al., 1993; Jiang et al., 2004). MAT-LyLu cells were harvested from sub-confluent culture by a brief exposure to 0.25% trypsin and 0.02% EDTA. After neutralizing trypsin with 10% FBS, the cells were washed, counted and resuspended in PBS containing SAC-Par-4-GFP or VC protein at a concentration of 20 μg/100 μl. All the tubes had a concentration of 0.5 × 10^6^ viable cells/100 μl. Before injecting the cells into the animals, all the cells were incubated at room temperature for 30 min. To avoid potential bias, the tubes containing cells (treated with different proteins) were coded as T1 (SAC-Par-4-GFP) and T2 (VC) and injected by an individual unaware of the nature of samples. Aliquots of 100 μl of cell suspension (0.5 × 10^6^ cells) were injected subcutaneously into the flank region of six different male Copenhagen rats (*n* = 3 for each group). Measurement of the tumor volumes in each rat were conducted between 10th day and 18th day post-injection. Tumor volume was calculated by using formula *V* = (*W*^2^ × *L*)^∗^0.52 for caliper measurements, where *V* is tumor volume, *W* is tumor width, and *L* is the tumor length. On day 18 post injection, all the animals were sacrificed for evaluation. Following this, tissues were preserved in 10% formalin, embedded in paraffin, sectioned (5 μm in thickness) and subjected to hematoxylin and eosin (H&E) staining (Sahoo et al., 2008).

### Statistical Analysis

Statistical analysis of all the data were performed adopting Student’s *t*-test (using Graph Pad Prism version 5.01) and presented as a mean of three or four independent experiments. The *p*-value of less than 0.05 was considered significant.

## Results

### Design and Construction of *In Planta* Expression Cassette and Generation of SAC-Par-4-GFP Expressing Transgenic Tobacco Plants

We designed two primary plant expression cassettes namely pKM24-SAC-Par-4-GFP and pKM24-GFP for this study. The pKM24-GFP was used as vector control (VC) while the other directs the expression of *SAC-Par-4-GFP* in plants under the control of the modified full-length M24 transcript promoter of the *Mirabilis mosaic virus* (**Figure 1**). The pKM24-GFP and pKM24-SAC-Par-4-GFP were used to generate transgenic plants (*Nicotiana tabacum* cv. Samsun NN).

### Analysis of Transgenic Tobacco Plants

**FIGURE 2:**
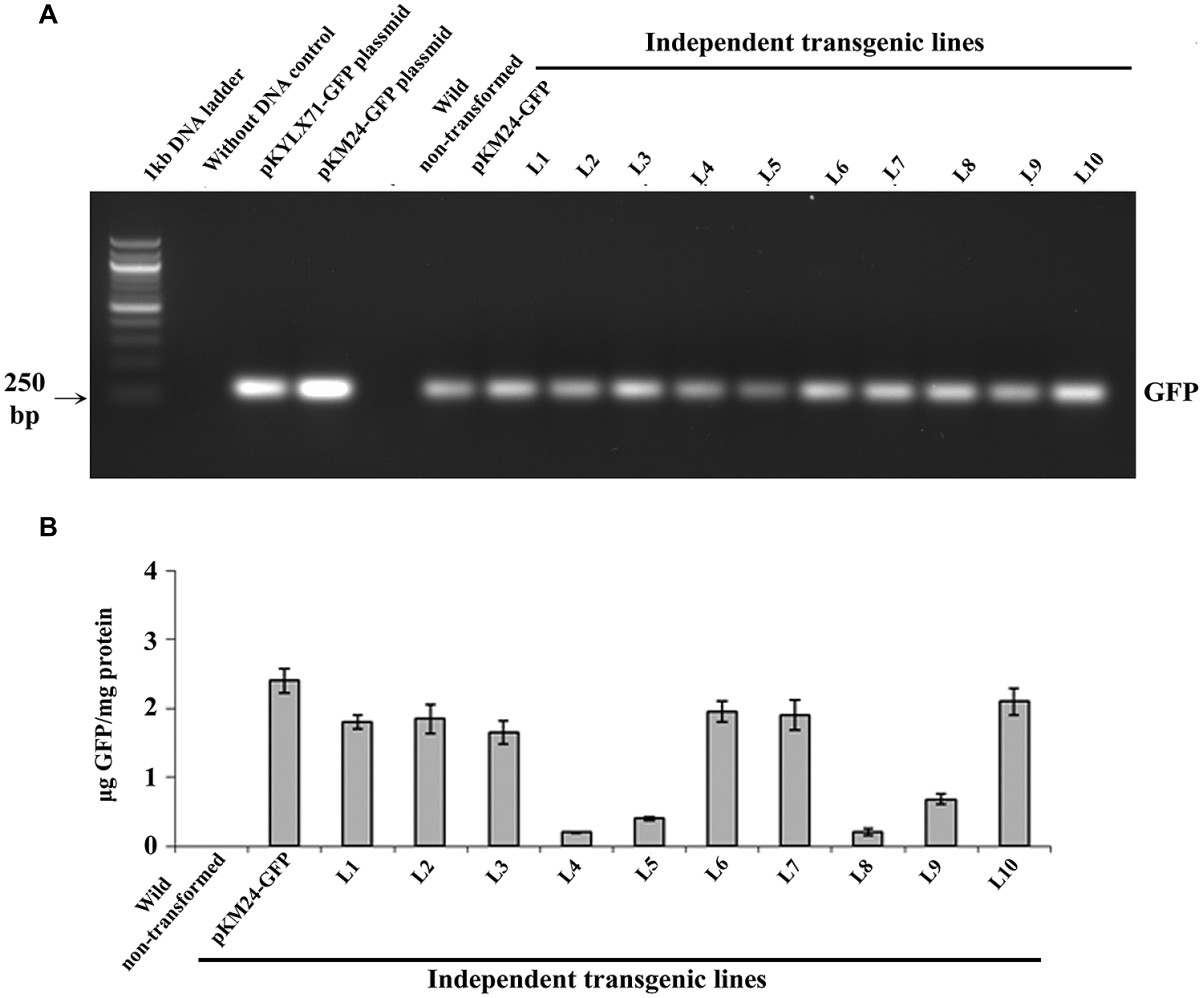
**Green Fluorescent Protein (GFP) analyses in transgenic tobacco lines. (A)** PCR amplification of *GFP* (250 bp) using genomic DNA isolated from 10 independent transgenic plants (T_2_ generation) expressing *SAC-Par-4-GFP*, vector control (VC) plant and wild-type plant; **(B)** The GFP protein concentration (expressed as μg GFP/mg protein) was measured in total soluble protein of transgenic and wild-type plants using Turner Biosystems Luminometer at GFP-UV module. Error bars indicate the standard deviation of readings from five different plants of each line (three readings per plant).

*GFP*-integration in all 10 independent pKM24-GFP and pKM24-SAC-Par-4-GFP T_2_ transgenic plants was confirmed by *GFP* (250 bp) amplification using PCR as described earlier (**Figure 2A**). Further, we detected enhanced GFP protein accumulation in the transgenic lines L1, L2, L3, L6, L7, and L10 (**Figure 2B**). Independent transgenic lines L2, L3, L5, and L6 were selected for further analysis. In this study, wild-type plants and the vector control-GFP plants were used as controls.

**FIGURE 3:**
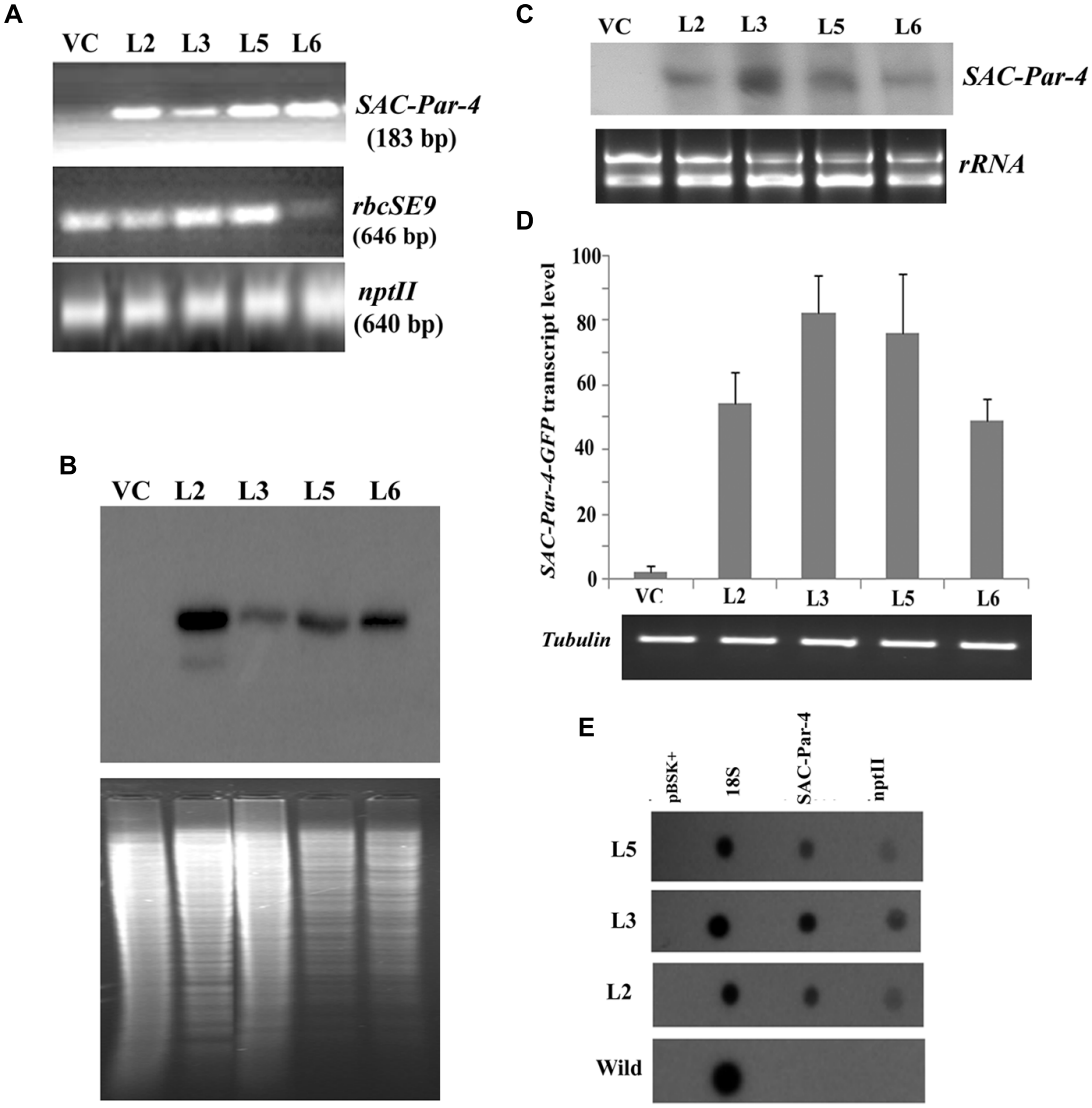
**Transgenic analyses. (A)** PCR amplification of different genes, viz. *SAC-Par-4* (i); *rbcSE9* (ii) and *npt*II (iii) from transgenic lines VC, L2, L3, L5, and L6 on 1.5% agarose gel; **(B)** Southern blot analysis to investigate *SAC-Par-4* gene insertion in transgenic plant lines VC, L2, L3, L5, and L6. Underneath is the agarose gel picture showing *Xho*I digested genomic DNA (10 μg) of T_2_ generation of four transgenic (L2, L3, L5, and L6 of SAC-Par-4-GFP) and vector control lines (prior blotting); **(C)** Northern blot analysis of *SAC-Par-4* mRNA expression in transgenic plants VC, L2, L3, L5, and L6. Underneath is an ethidium bromide-stained gel showing *rRNA* quality; **(D)** Real-time analysis of *SAC-Par-4* transcripts in VC, L2, L3, L5, and L6 lines. The data were normalized by *tubulin* transcripts. The data shown are mean values from three independent experiments. Bars indicate the standard errors of means; **(E)** Nuclear run-on assay of *SAC-Par-4* transcripts. Dot blots were hybridized to ^32^P-labeled nascent transcripts from wild-type plant and transgenic lines L2, L3, and L5 which were synthesized by run-on transcription. pBSK plasmid (1 μg) and 18S ribosomal DNA (0.5 μg) were used as controls.

Furthermore, we confirmed the integration of 183 bp long *SAC-Par-4* in L2, L3, L5, and L6 transgenic pKM24-SAC-Par-4-GFP plant but not in pKM24-GFP (VC) plant (**Figure 3A**). We also demonstrated integrations of different components of expression cassette by PCR- amplification of *rbcSE9* and *npt*II in the above plant lines (**Figure 3A**).

For Southern blot analysis, the genomic DNA from transgenic lines L2, L3, L5, L6, and VC was digested with the restriction enzyme *Xho*I. The selection of this enzyme is based on the fact that it digests the genomic DNA with reasonable frequency, but acts as a single cutter for transgene construct (not inside the *SAC-Par-4* gene; **Figure 1B**). Southern analysis confirmed integration of the *SAC-Par-4* transgene in the genome of L2, L3, L5, and L6 transgenic lines; we observed higher copy number for L2 line in comparison to the other lines. Overall, appearance of discrete and alike Southern positive bands (from different transgenic lines) suggests that integration of *SAC-Par-4* is in tandem fashion in plant genome, indicating independent nature of transgenic events (**Figure 3B**).

Alongside, Northern analysis, as describes earlier, yielded a fair signal for the presence of *SAC-Par-4* transcripts in L2, L3, L5, and L6 lines harboring pKM24-SAC-Par-4-GFP, while no signal was detected in pKM24-GFP plant. We observed variations in signal intensities of Northern bands among different lines (**Figure 3C**); with highest expression in line L3. This observation support the data obtained from Real-time RT-PCR experiment. We observed expression of *SAC-Par-4-GFP* transcripts in following order: L3 > L5 > L2 > L6 (**Figure 3D**).

We performed nuclear run-on assays using intact nuclei isolated from L2, L3, and L5 lines to measure the accumulation of *SAC-Par-4-GFP* transcripts *in vivo* and to analyze potential differences in the transcription initiation between the transgenic lines. Data obtained revealed that *SAC-Par-4, npt*II, and *18S* genes were expressed in above lines, with the strongest transcription rate found in L3 line which is supporting the result obtained from Northern blot and Real-time assays (**Figure 3E**).

### ELISA and Western Blot Analysis of Plant-derived SAC-Par-4-GFP

**FIGURE 4:**
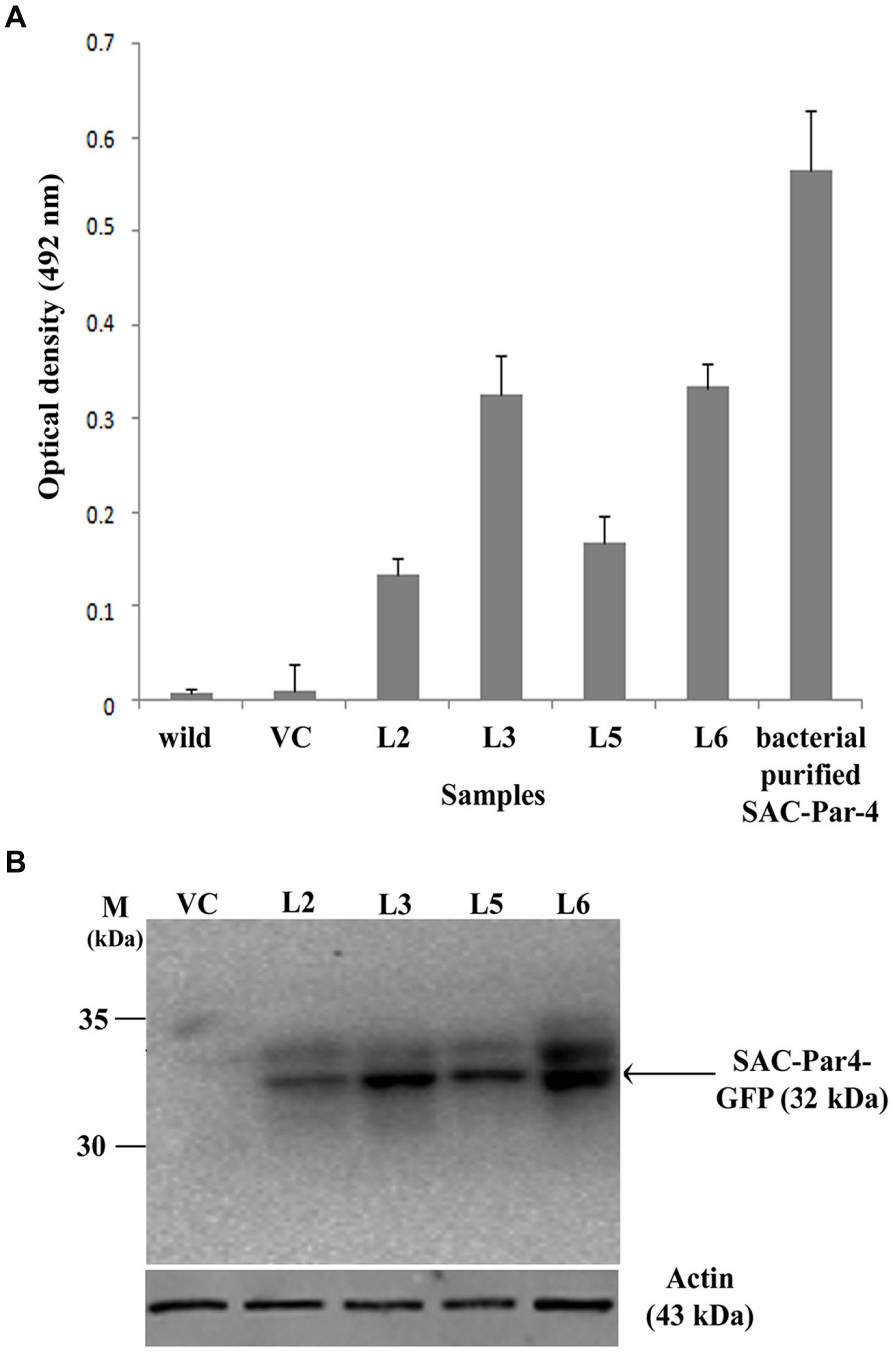
**Evaluation of recombinant SAC-Par-4-GFP accumulation. (A)** ELISA analysis of recombinant SAC-Par-4-GFP accumulation from L2, L3, L5, and L6 transgenic plants along with wild-type and VC plant. Values are the average of three independent experiments. Bars indicate standard deviations; **(B)** Detection of SAC-Par-4-GFP protein on the Western blot from different transgenic lines, indicated by arrow. Numbers on the left indicate the size of protein standard. β-actin was used as loading control.

The accumulation level of recombinant SAC-Par-4-GFP in different transgenic lines (L2, L3, L5, and L6) was evaluated by ELISA as described earlier. We noticed SAC-Par-4-GFP accumulation levels varied among independent transgenic plants (T_2_) ranging from 0.05% to 0.15% total soluble protein or 1.6–10.5 μg/g fresh leaf weight (results not shown in detail). The L3 line displayed maximum recombinant SAC-Par-4-GFP accumulation (0.15%) as compared to L2, L5, and L6 (**Figure 4A**). The expression of ER-targeted SAC-Par-4-GFP-SEKDEL in infiltrated leaves reached the highest level at day 3 post-infiltration (dpi), with yields upto 88 μg/g fresh weight.

In continuation, we carried out Western blot analysis to check the integrity of the plant-derived SAC-Par-4-GFP as described earlier and observed a band of around 32 kDa SAC-Par-4-GFP protein on the blot from L2, L3, L5, and L6 tobacco plants (**Figure 4B**).

### Deglycosylation Analysis of Plant-derived SAC-Par-4-GFP

**FIGURE 5:**
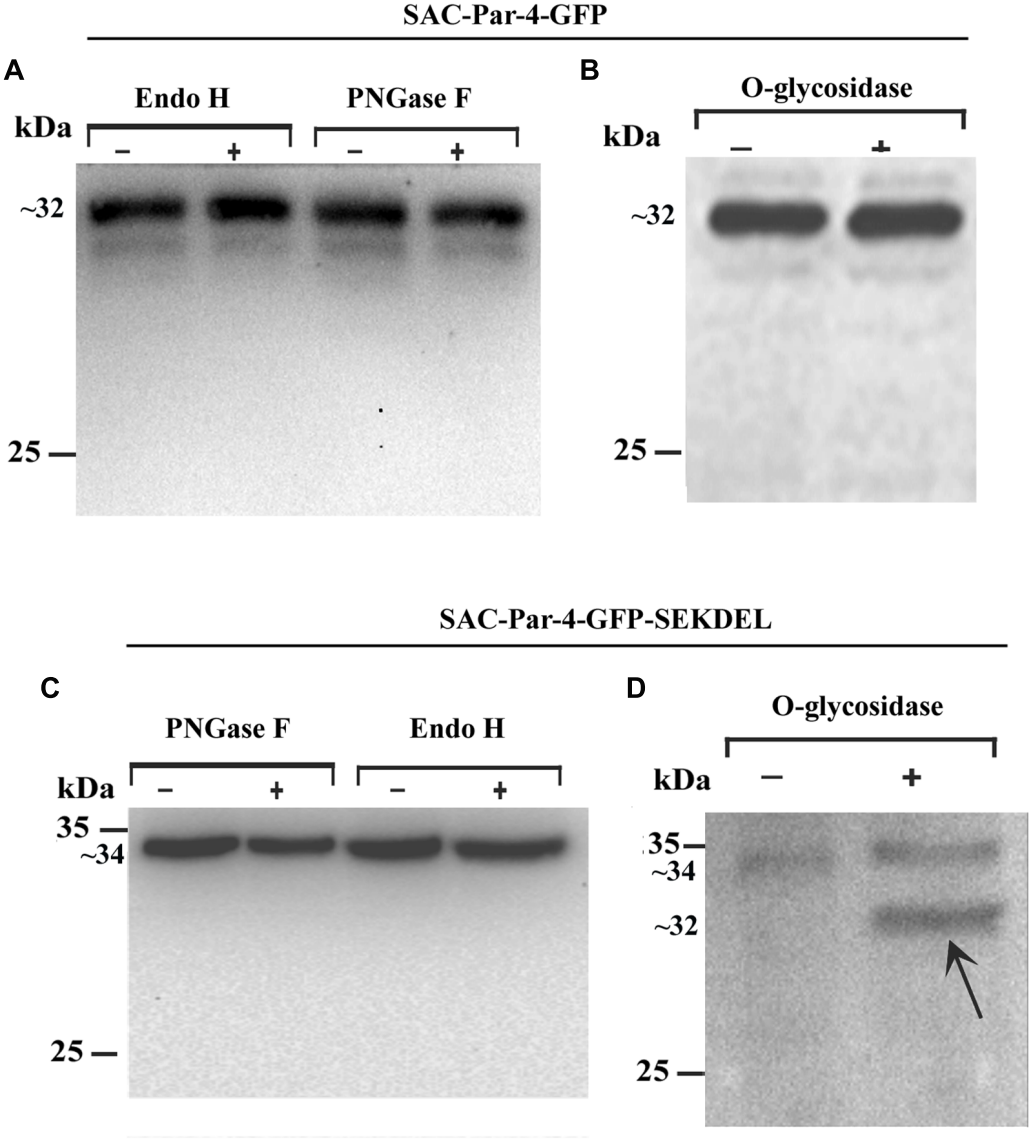
**Deglycosylation analysis of recombinant apoplast-targeted SAC-Par-4-GFP of transgenic line L3 and ER-targeted SAC-Par-4-GFP-SEKDEL of agroinfiltrated tobacco leaves protein extract.** Western blot analysis of **(A)** SAC-Par-4-GFP protein treated with endo-β-*N*-acetylglucosaminidase H (Endo H) and peptide-*N*-glycosidase F (PNGase F); Western blot analysis of **(B)** *O*-glycosidase treated protein extract obtained from transgenic SAC-Par-4-GFP. Numbers on the left indicate the positions of protein size markers. “+” indicates in presence of enzymes and “–” indicates without enzymes. Western blot analysis of **(C)** SAC-Par-4-GFP-SEKDEL protein treated with peptide-*N*-glycosidase F (PNGase F) and endo-β-N-acetylglucosaminidase H (Endo H; left); Western blot analysis of **(D)** *O*-glycosidase treated protein extract obtained from transiently expressed SAC-Par-4-GFP-SEKDEL. Arrows point to the deglycosylated form of plant SAC-Par-4. Numbers on the left indicate the positions of protein size markers.

We performed deglycosylation assays on the protein extracts obtained from transgenic line L3 expressing SAC-Par-4-GFP and found that the apoplast-targeted SAC-Par-4-GFP was fully resistant to endoglycosidase H (EndoH) digestion (**Figure 5A**, lanes 1 and 2) and peptide *N*-glycosidase F (PNGaseF) treatment (**Figure 5A**, lanes 3 and 4), suggesting that SAC-Par-4-GFP does not have any glycan molecules sensitive to both of these enzymes. In parallel, the efficacies of these enzymes (Endo H and PNGase F) were validated by digestion of a different plant- derived protein namely human peroxisomal Δ^2^,Δ^3^-enoyl CoA isomerase protein HsPECI2 (Rai et al; unpublished data from our group). Furthermore, enzymatic treatment with α-neuraminidase and *O*-glycosidase followed by Western blot analysis with anti-PAR-4 polyclonal antibody displayed no band shift for apoplast-targeted SAC-Par-4-GFP protein extract demonstrating that SAC-Par-4-GFP was resistant to *O*-glycosidase treatment (**Figure 5B**). Taken together, these results indicate plant-derived apoplast-targeted SAC-Par-4-GFP is not glycosylated.

Furthermore, deglycosylation analysis was performed with the total protein extracts obtained from agroinfiltrated tobacco leaves expressing ER-targeted SAC-Par-4-GFP-SEKDEL as described in the Section “Material and Methods.” Data obtained revealed plant-derived SAC-Par-4-GFP-SEKDEL was resistant to both PNGase F and Endo H (**Figure 5C**). Interestingly, *O*-glycosidase treatment followed by Western blot analysis displayed a new smaller band with a molecular mass of approximately 32 kDa in *O*-glycosidase treated SAC-Par-4-GFP-SEKDEL protein extract compared to the untreated sample which migrated around 34 kDa (**Figure 5D**). Hence, the SEKDEL-tagged SAC-Par-4-GFP proteins were sensitive to *O*-glycosidase, suggesting presence of *O*-linked glycans indicative of ER localization with efficient retention or retrieval from the *cis*-Golgi.

### Proteolytic Stability of Plant-derived SAC-Par-4-GFP

**FIGURE 6:**
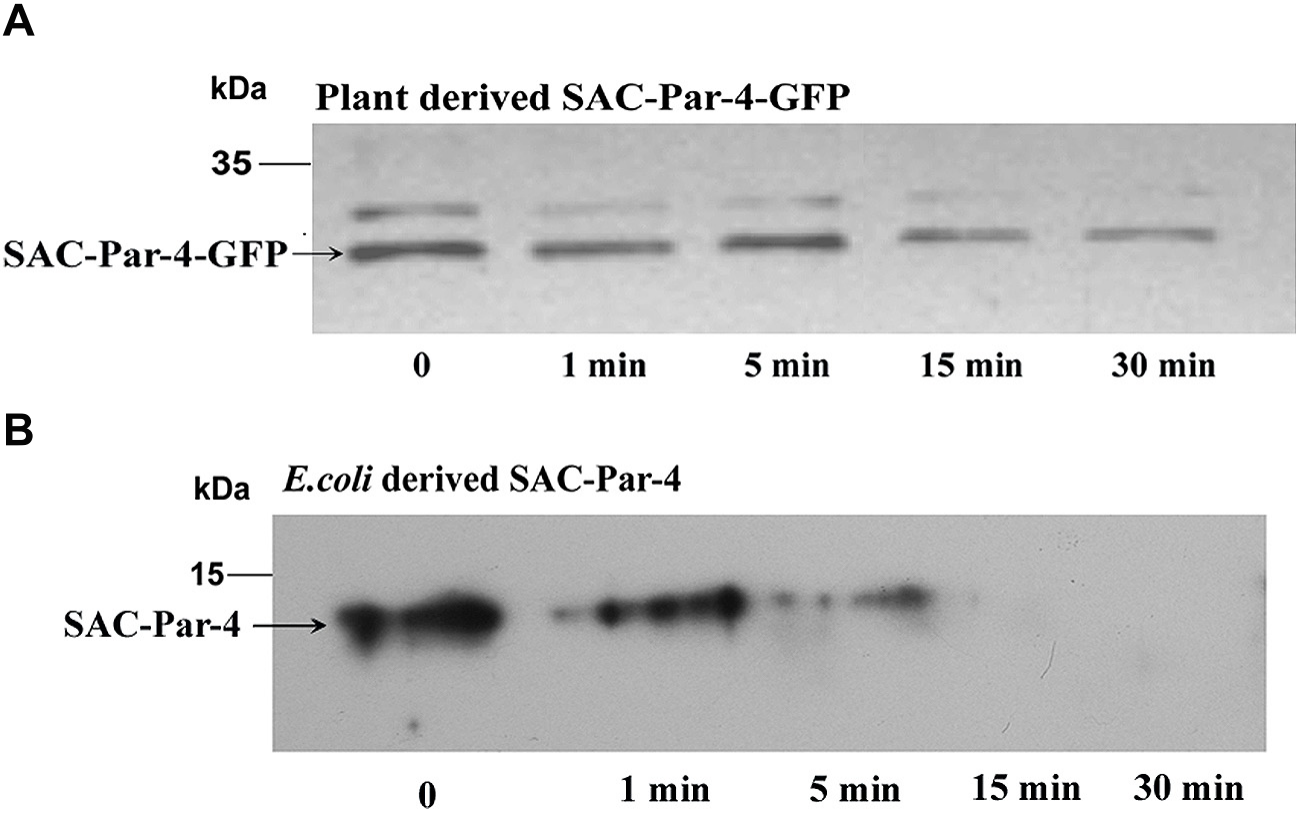
**Proteolysis assay.** Trypsin digestion of recombinant SAC-Par-4 protein for various time increments and Western blot analysis using anti-Par-4 antibody of **(A)** apoplast-targeted SAC-Par-4-GFP protein of transgenic line L3; **(B)** bacterial derived SAC-Par-4. Each lane contains the starting equivalent of approximately 500 ng total protein. The position of the molecular weight marker (M) is indicated.

Trypsin digestions were carried out with total protein extract obtained from plant-derived SAC-Par-4-GFP, SEKDEL-tagged SAC-Par-4-GFP and bacterial purified SAC-Par-4 as described in Section “Material and Methods” (**Figure 6** and Supplementary Figure S2). To evaluate the resistance or sensitivity of these proteins toward trypsin digestion, Western blot analysis was performed with anti-Par-4 antibody by taking various aliquots of the trypsin-treated protein extracts at different time points (0, 1 min, 5 min, 15 min, and 30 min). The applied trypsin ratio to recombinant proteins was 40:1 and 10:1 (w/w) for plant extract and bacterial purified protein, respectively; Result of **Figure 6** confirmed that plant-derived SAC-Par-4-GFP was resistant to trypsin digestion even after 15 min post-treatment while *Esc*herichia *coli* derived SAC-Par-4 got degraded within 10 min of incubation. Furthermore, approximately 90% of SAC-Par-4-GFP-SEKDEL remained intact after 30 min of incubation (Supplementary Figure S2). The enhanced stability of ER-targeted SAC-Par-4-GFP-SEKDEL could be attributed to the fact that this protein is getting effectively glycosylated. This result demonstrated that plant-derived SAC-Par-4-GFP showed enhanced protection to trypsin digestion.

### Purification of Plant-derived SAC-Par-4-GFP Protein

**FIGURE 7:**
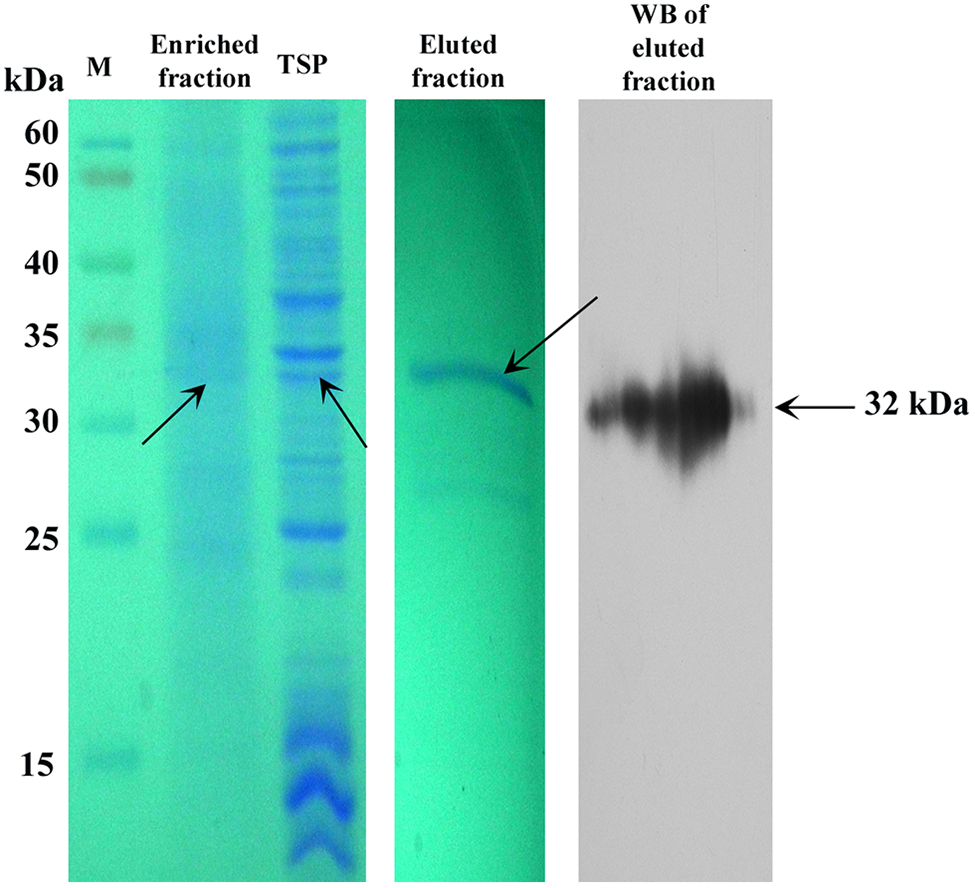
**Purification of plant-derived SAC-Par-4-GFP.** Coomassie-stained 10% SDS-PAGE gel and Western blot showing the partial purification of SAC-Par-4-GFP from the pKM24-SAC-Par-4-GFP transformed line L3 along with the enriched fraction. Total soluble protein and eluted fraction from affinity purification are shown. Arrows indicate molecular size.

We partially purified SAC-Par-4-GFP from transgenic line L3 by antibody pull-down approach, as described in “Material and Methods” section. The enrichment coupled with affinity chromatography process exhibited 50% recovery and above 70% purification of the SAC-Par-4-GFP as confirmed by SDS-PAGE and Western blot (**Figure 7**).

### Plant-derived SAC-Par-4-GFP Inhibited Cell Growth, Induced Apoptosis *In Vitro* and Delayed the Onset of Tumor Growth *In Vivo*

**FIGURE 8:**
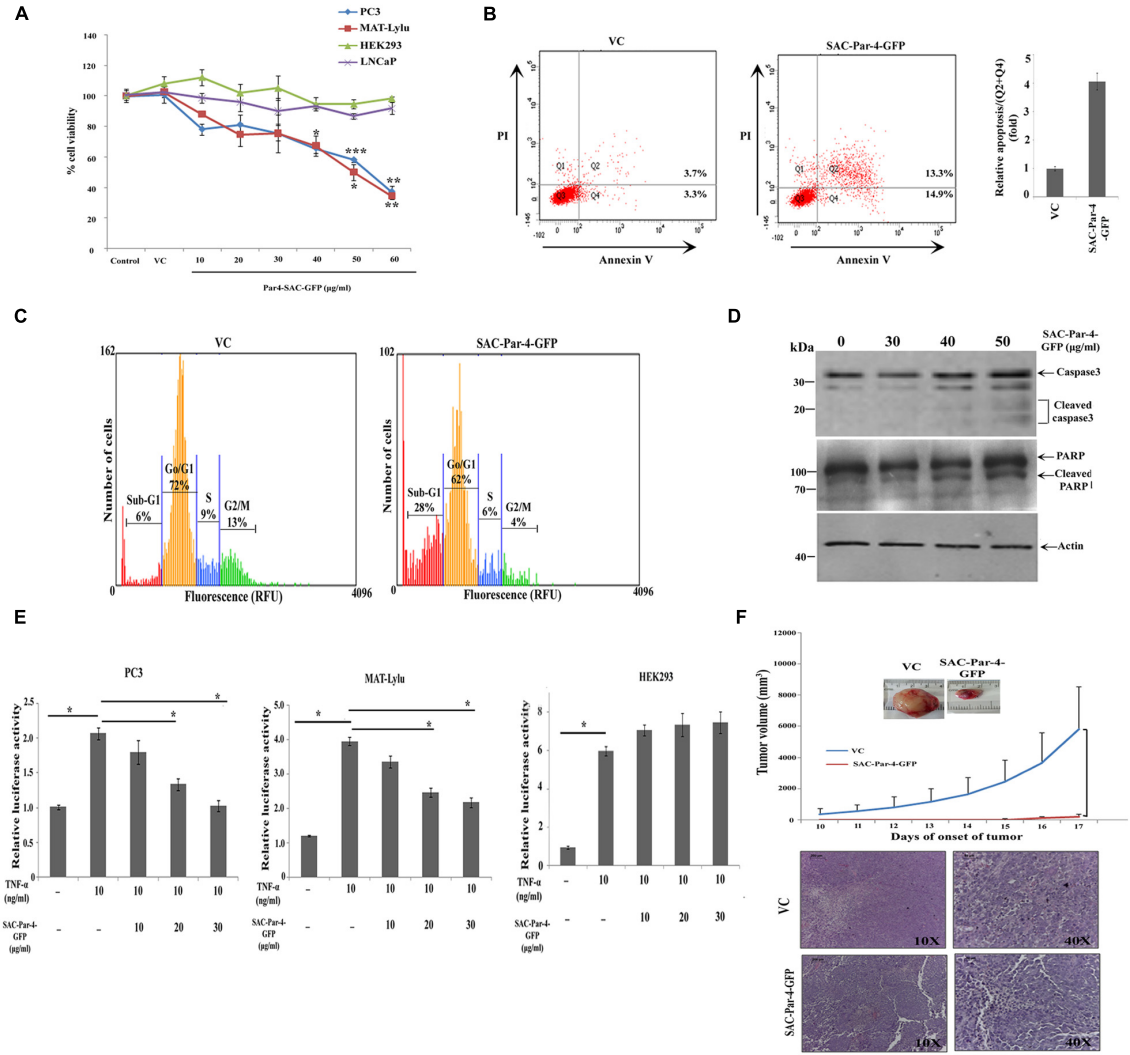
**Bioactivity of plant-derived SAC-Par-4 *in vitro* and *in vivo*. (A)** PC3, MAT-LyLu, LNCaP, and HEK293 cells were seeded in 96 well plate, incubated overnight and treated accordingly with vector control protein (100 μg/ml) or different concentrations (10, 20, 30, 40, 50, and 60 μg/ml) of SAC-Par-4-GFP protein for 48 h. Cell viability was determined by MTT assay and normalized to control for each cell line. The data represented as a mean of three independent experiment, ±SD, *n* = 3, ^∗^*p* < 0.05, ^∗∗^*p* < 0.005 and ^∗∗∗^*p* < 0.001; **(B)** PC3 (1 × 10^6^ cells) were treated with vector control protein (100 μg/ml) and partially purified SAC-Par-4-GFP protein (50 μg/ml), and the percentage of apoptotic cells was analyzed by AnnexinV-FITC/PI staining. Experiment was repeated two times and quantitative result (Q2 + Q4) is displayed as the mean fold change (±SD) compared with control from two independent experiments; **(C)** Cell cycle analysis of PC3 cells treated with vector control protein (100 μg/ml) and SAC-Par-4-GFP protein (50 μg/ml). Data are representative of three independent cell cycle analyses and expressed as a percentages of cells found in different cell population, sub-G_1_ (apoptotic cells), G_0_/G_1_, S, and G_2_/M; **(D)** Immunoblot analysis demonstrates cleaved caspase-3 and cleaved PARP expression in PC3 cells treated with different concentration of SAC-Par-4-GFP protein for 48 h; **(E)** PC3-NF-κB-luc, MAT-LyLu-NF-κB-luc, and HEK293-NF-κB-luc reporter cells were treated with TNFα (10 ng/ml) or SAC-Par-4-GFP protein in concentrations of 10, 20, and 30 μg/ml for 6 h. The results were represented as change in relative luciferase activity, ±SD of three independent experiments and ^∗^*p* < 0.05; **(F)** MAT-LyLu cells pre-treated and co-injected with SAC-Par-4-GFP protein or vector control protein (20 μg in 100 μl cell suspension) were injected into the flank region of male Copenhagen rats (*n* = 3 for each group). After 10 days, tumor volumes were measured every day and plotted. Points in graphics are volume mean values and standard deviations. H&E staining confirmed presence of viable tumor cells in all the isolated tumor tissues.

The biological activity of plant-derived partially purified SAC-Par-4-GFP from L3 line was determined by MTT assay on PC3, MAT-LyLu, and LNCaP cell lines at different concentrations (10, 20, 30, 40, 50, and 60 μg/ml) for 48 h as described earlier. The viability of PC3 and MAT-LyLu cells were reduced with increasing concentration of SAC-Par-4-GFP protein, and maximum cell death was observed up to 63 and 66%, respectively, at 60 μg/ml concentrations (**Figure 8A**). Further, the IC_50_ values were found to be 43 μg/ml and 50 μg/ml for PC3 and MAT-LyLu cells, respectively. However SAC-Par-4-GFP exhibited maximum 10–15% growth inhibition in LNCaP cells. On the other hand, the protein obtained from pKM24-GFP (VC) showed no cytotoxic effect at 100 μg/ml concentration. In parallel, we observed plant-derived SAC-Par-4-GFP exhibited no cytotoxicity on HEK293 cell line, which is non-cancerous in nature (**Figure 8A**).

We performed AnnexinV-FITC/PI staining to investigate whether SAC-Par-4 induces apoptosis in the PC3 cancer cell and non-cancerous HEK293. As shown in **Figure 8B**, treatment with 50 μg/ml of SAC-Par-4-GFP for 48 h induced apoptosis in PC3 cells and increased the percentage of early and late apoptotic cells by approximately fourfold compared to the vector control. At the same time, in case of HEK293 cell line, there was no significant difference between the total percentages of apoptotic cells in vector control and SAC-Par-4-GFP treated cells (data not shown). Further cell cycle analysis in PC3 cells treated with SAC-Par-4-GFP protein (50 μg/ml) for 48 h revealed significant increase in sub-G_1_ population (sub-diploid DNA fraction) by 4.6 fold compared to the vector control and this population represented the apoptotic portion, implying SAC-Par-4 promote apoptosis (**Figure 8C**). In the same direction, we observed signals for apoptotic markers like cleaved caspase-3 and cleaved PARP in PC3 cells treated with 50 μg/ml of purified SAC-Par-4-GFP for 48 h (**Figure 8D**).

Suppression of NF-κB activity by SAC-Par-4 in PC3-NF-κB-luc and MAT-LyLu-NF-κB-luc cells was studied as described earlier. NF-κB luciferase activity was significantly increased in PC3 cells by approximately twofold and in MAT-LyLu cells by approximately fourfold in TNF-α-stimulated cells compared with the untreated control group. However, the TNF-α-stimulated luciferase activity was significantly reduced (*p* < 0.05) to approximately twofold by 30 μg/ml of SAC-Par-4 treatment in both cell lines. In parallel, there was no suppression of NF-κB activity observed in case of TNF-α-stimulated HEK293- NF-κB-luc cells upon treatment with 30 μg/ml of SAC-Par-4-GFP (**Figure 8E**).

In the *in vivo* study, we have examined the effect of plant-derived affinity-purified SAC-Par-4 on tumor incidence in a syngenic rat prostate cancer model. The result from this investigation indicated that SAC-Par-4-GFP pre-treatment and co-injection with the MAT-LyLu cells inhibited the rate of tumor growth *in vivo* as compared to control group. Visible tumors appeared at sixth day after the injection of cells pre-treated with pKM24-GFP vector control protein. Whereas, there was no visible tumor up to 14th day in animals injected with SAC-Par-4-GFP pre-treated cells. Moreover, tumor volume measured after the 10th day of cancer cells and vector control protein injection showed exponential growth of all the tumors of the control animals. Further the hematoxylin and eosin (H&E) staining confirmed the presence of viable cancer cells in all tumor tissues; alongside the staining ruled out the possibility of non-tumor (necrotic) mass (**Figure 8F**).

## Discussion

There are several definite advantages of using plants as bioreactor for large-scale production of recombinant biologics at cheaper rate and they are capable of imparting post-translational modification in synthesized protein. We raised transgenic tobacco plants expressing codon optimized rat *SAC-Par-4-GFP* gene under the control of strong constitutive M24 promoter; a constitutive promoter with 25-fold stronger activity than the CaMV35S promoter (Dey and Maiti, 1999a,b). We deliberately fused a translational enhancer sequence (5′ AMV) and apoplast targeting sequence (aTP) of *Arabidopsis* 2S2 protein gene to ensure stability and enhanced production of SAC-Par-4 (Schillberg et al., 1999; Benchabane et al., 2008), (**Figure 1**). Additionally, for the transient assay we coupled the SEKDEL signal sequence to the C-terminus of the SAC-Par-4-GFP to target the recombinant SAC-Par-4-GFP to the ER lumen for proper post-translational modifications, such as glycosylation and disulphide bond formation (Supplementary Figure S1; Hwang et al., 1992; Ma et al., 2005b; Wang et al., 2008).

Rate of seed germination, segregation analysis (**Table 2**), molecular characterization, gene integration and expression analysis of SAC-Par-4-GFP transgenic lines were performed (**Figures 2–4**); based upon the GFP content in the above lines, we have selected three lines (L2, L3, and L6) with higher GFP accumulation and one with low GFP content L5 for further study. The higher accumulation of plant-derived SAC-Par-4-GFP may be attributed to several factors acting in synergy, including the stable nature of the protein itself, the use of a strong M24 promoter coupled with a 5′ AMV–2S2 signal peptide sequence. We found a strong expression of SAC-Par-4-GFP both at transcript and protein levels in L3 transgenic plant, while moderate to weak expression in L2, L5, and L6 transgenic plants, respectively. Even though, we noted higher copy number shown by signal obtained in Southern hybridization for L2 line compared to L3 line, the integration at the heterochromatin region of the tobacco genome might implicate the silencing of *SAC-Par-4-GFP* in L2 line as reported earlier by Xu-Gang et al. (2002). Furthermore, we found strong GFP expression at the periphery of guard cells and trichome stalk cell of apoplast targeted SAC-Par-4-GFP in transgenic line L3 (Supplementary Figure S3). In lieu of these observations, we finally selected L3 line for further Affi-gel Par-4 based affinity purification of SAC-Par-4-GFP.

**Table 2:** Segregation Analyses.

Plant lines	Segregation (Kan^R^:Kan^S^)	% germination	Showing 3:1 segregation
pKM24-SAC-Par-4-GFP#1	38R:14S (2.7:1) 30R:12S (2.5:1) 41R:11S (3.7:1)	100% 92% 100%	Yes
pKM24-SAC-Par-4-GFP#2	37R:13S (2.8:1) 32R:11S (2.9:1) 38R:16S (2.37:1)	98% 100% 96%	Yes
pKM24-SAC-Par-4-GFP#3	41R:15S (2.73:1) 34R:14S (2.43:1) 34R:13S (2.61:1)	94% 100% 98%	Yes
pKM24-SAC-Par-4-GFP#4	40R:12S (3.33:1) 38R:16S (2.25:1) 40R:9S (4.44:1)	84% 82%	Yes
pKM24-SAC-Par-4-GFP#5	32R:21S (1.52:1) 26R:16S (1.62:1)	46%	No
pKM24-SAC-Par-4-GFP#6	33R:13S (2.54:1) 39R:14S (2.78:1)	100% 96% 92%	Yes
pKM24-SAC-Par-4-GFP#7	27R:12S (2.25:1) 38R:12S (3.2:1) 34R:12S (2.83:1)	94% 100% 92%	Yes
pKM24-SAC-Par-4-GFP#8	27R:19S (1.42:1) 32R:21S (1.52:1)	74%	No
pKM24-SAC-Par-4-GFP#9	26R:21S (1.24:1) 23R:14S (1.64:1)	76%	No
pKM24-SAC-Par-4-GFP#10	35R:13S (2.69:1) 40R:12S (3.33:1) 36R:14S (2.57:1)	100% 94% 100%	Yes

The major advantage of using plant as a bioreactor is that it glycosylates the recombinant protein along the secretory pathway as proteins move from the ER through the Golgi to their final destination. Therefore, the nature of glycosylation and proteolytic stability of the apoplast-targeted SAC-Par-4-GFP and ER-targeted SAC-Par-4-GFP-SEKDEL protein were analyzed (**Figure 5**). Glycosylation enhances the physiochemical properties of a protein by endeavoring thermal resistance, protection from proteolytic degradation and stability (Ma et al., 2005c). To attest this preposition, we targeted SAC-Par-4-GFP protein to the secretory pathway using apoplast signal sequence and ER-specific SEKDEL sequence and performed deglycosylation assays as described previously. Our results indicated that the plant-derived SAC-Par-4-GFP may contain complex-type N and *O*-glycans which were inaccessible to the deglycosylating enzymes Endo H, PNGaseF, and *O*-glycosidase which is in accordance to a previous report for a different plant-derived protein namely human erythropoietin, EPO (Conley et al., 2009). In contrast, the SEKDEL-tagged SAC-Par-4-GFP proteins were sensitive to *O*-glycosidase (**Figure 5D**) unlike the apoplast-targeted SAC-Par-4-GFP (**Figure 5B**), suggesting presence of *O*-linked glycans in ER-targeted SAC-Par-4-GFP-SEKDEL. Non-glycosylated plant-derived SAC-Par-4-GFP had almost similar molecular mass (∼32 kDa) to that of deglycosylated SAC-Par-4-GFP-SEKDEL (∼32 kDa) and had lower molecular weight to that of the glycosylated SAC-Par-4-GFP-SEKDEL (∼34 kDa). This is most likely because of the addition of the ER retention signal motif SEKDEL to the C-terminus of plant-derived SAC-Par-4-GFP along with *O*-linked oligosaccharide chain (Conley et al., 2009). *In silico* analysis of SAC-Par-4-GFP-SEKDEL using NetOGlyc 4.0.0.13 software^2^ affirmed two potential *O*-glycosylation sites at positions 20 and 21 located in SAC-Par-4-GFP. Over and above the recombinant SAC-Par-4-GFP and SAC-Par-4-GFP-SEKDEL protein showed resistance to trypsin digestion for 15–30 min compared to bacterial derived SAC-Par-4 (**Figure 6** and Supplementary Figure S2). This could be explained by the fact that plant-derived SAC-Par-4 in fusion with another protein, GFP, is protected against trypsin digestion as compared to that of bacterial SAC-Par-4. Besides this, effective glycosylation of SAC-Par-4-GFP-SEKDEL protein and its enhanced stability could be the other reasons for increasing the therapeutic value of plant derived SAC-Par-4-GFP-SEKDEL protein. To achieve higher and more stable expression, we have begun to produce transgenic tobacco lines homozygous for the introduced *SAC-Par-4-GFP-SEKDEL* gene.

The cytotoxic effect of affinity-purified SAC-Par-4 on PC3 and MAT-LyLu cells indicates that plant-derived SAC-Par-4 retains its biological activity (**Figure 8A**). Importantly, its effect on both the cancer cells but not on HEK293 cells suggests its specificity against prostate cancer. Furthermore, the precise role of plant-derived SAC-Par-4 as a potent anti-prostate cancer agent needs to be evaluated in reference to its activity against normal prostate cell lines such as PNT1 A/B or PNT2. An earlier report (Burikhanov et al., 2009) indicated that normal/immortalized cells fail to respond to exogenous SAC-Par-4 due to their lower content of specific cell surface receptor GRP78 in addition to lack of robust ER-stress response. Moreover, human embryonic kidney cell line HEK293 has been used as a representative normal cell line in multiple studies (Imberg-Kazdan et al., 2013; Zhang et al., 2014; Al-Sheddi et al., 2015; Kim et al., 2015). HEK293 cells being immortalized and non-cancerous in nature, have lower expression levels of GRP78 in comparison to cancer cells which justifies its inclusion as appropriate control in this study (Li et al., 2008; Burikhanov et al., 2009; Dai et al., 2010). Apart from evaluating the inhibitory activity of plant-derived SAC-Par-4 in androgen receptor negative cell lines like PC3 and MAT-LyLu, we also studied SAC/Par-4 effect in an AR positive prostate cell line (LNCaP). Plant-derived SAC-Par-4 showed very low inhibitory effect (10–15% sensitive) in LNCaP cells. This effect is not significantly higher than control treatment, which indicates that they could be resistant or less responsive to induction of apoptosis by plant-made SAC-Par-4. This is consistent with the findings of Chakraborty et al. (2001) where the authors described that *Par-4* is able to induce apoptosis in PC3, DU-145, and TSU-Pr cells, while LNCaP are resistant. Furthermore, the mechanism underlying the differential cytotoxic effect of plant-derived SAC-Par-4 on AR positive and AR negative human prostate cancer cells needs further investigation. The cytotoxic effect of this protein on both human (PC3) and rat prostate cancer cell lines (MAT-LyLu) further suggests its efficacy on cancer cells of different origin, and this might be due to the highly conserved SAC domain of Par-4 (El-Guendy and Rangnekar, 2003). A similar kind of effect of Par-4 on cancer cells of multi-species origin have also been reported earlier (Vetterkind et al., 2005; Shukla et al., 2013). To rule out the effect of other proteins derived from the different components of the vector and other soluble proteins of the plant, we compared the result obtained by purified SAC-Par-4 protein with the protein obtained from vector control plant. Our observations from Annexin-V/PI staining, cell cycle analysis and Western blotting in PC3 cell line (**Figures 8B–D**) confirms that plant-derived SAC-Par-4 is able to specifically kill cancer cell via apoptosis similar to that observed earlier (Burikhanov et al., 2009). Moreover, the NF-κb luciferase assay (**Figure 8E**) clearly demonstrates that plant-derived SAC-Par-4 is able to significantly suppress the NF-κb activity in both PC3 and MAT-LyLu cells which is in corroboration to the previous findings (El-Guendy et al., 2003; Zhao and Rangnekar, 2008).

In the present study, we checked the anti-tumor activity of plant-derived partially purified SAC-Par-4-GFP by injecting MAT-LyLu cells pre-treated and co-injected with SAC-Par-4-GFP or VC protein. We observed visible tumors in case of SAC-Par-4-GFP pre-treated cells on the 15th day of cells’ injection (*n* = 2/3). However, all the animals injected with VC pre-treated cells had a visible tumor by sixth day of cancer cells injection (*n* = 3/3; **Figure 8F**). The delayed onset in the growth of tumor in case of SAC-Par-4-GFP pre-treated MAT-LyLu cells might be due to the fact that pre-incubation with SAC-Par-4-GFP induced cell-death and at the same time the cells that escape the cytotoxic effect repopulated to make a visible tumor at the later time point. Although the animal number in this study is not so high but we noted a clear visible difference in the tumor volume developed between SAC-Par-4-GFP treated and VC protein treated rat groups. A similar animal cohort (*n* = 3) has also been used by other investigators in literature (Moore et al., 2001; Liu et al., 2014). These observations have encouraged us to check the efficacy of SAC-Par-4 protein in large animal cohort as a future prospect of this project. In this regard we have planned to exploit MAT-LyLu cells labeled with luciferase and implanted orthotopically in future.

*In vitro* and *in vivo* results of our present findings may be considered as a starting point for ascertaining the anti-prostate cancer activity of plant-derived recombinant SAC-Par-4. In order to effectively demonstrate the generalized cancer-specific efficacy of plant-isolated SAC-Par-4, more and different robust experiments are required to be performed. These include evaluation and comparative analysis of anti-tumorigenic activities of plant-derived SAC-Par-4 in several AR positive cell lines (LNCaP, LAPC4, and MDA PCa 2b), AR negative cell lines (PC3, MAT-LyLu, and DU145), normal prostate cell line (PNT1-A/B, PNT2PrE, and PrS), immortalized human prostate epithelial cells (PZHPV7) along with detailed studies in a large cohort of animals. Such vigorous approaches will be required for further establishing the anti-cancer effect of plant-derived SAC-Par-4 and also to investigate its possible undesired side-effects on normal cells.

Taken together, in the current study we have engineered plant for efficient production and isolation of plant-derived SAC-Par-4-GFP protein. Importantly, this protein is biologically active and able to reduce the growth of the prostate cancer cell lines (PC3 and MAT-LyLu). This cytotoxic effect is potentially through NF-κb suppression and induction of apoptosis. Our *in vitro* and *in vivo* studies suggest the potential of plant-derived SAC-Par-4-GFP as a therapeutic agent against prostate cancer cells; however, further extensive studies are essential to evaluate the efficacy of this protein in a more clinically relevant model.

## Conflict of Interest Statement

The authors declare that the research was conducted in the absence of any commercial or financial relationships that could be construed as a potential conflict of interest.

## Footnotes

1 http://www.lifetechnologies.com/GeneArt/

2 http://www.cbs.dtu.dk/services/NetOGlyc/

## References

[B1] Acharya S. Sengupta S. Patro S. Purohit S. Samal S. K. Maiti I. B. (2014). Development of an intra-molecularly shuﬄed efficient chimeric plant promoter from plant infecting Mirabilis mosaic virus promoter sequence. *J. Biotechnol.* 169 103–111. 10.1016/j.jbiotec.2013.08.022 24060830

[B2] Allen G. C. Flores-Vergara M. A. Krasynanski S. Kumar S. Thompson W. F. (2006). A modified protocol for rapid DNA isolation from plant tissues using cetyltrimethylammonium bromide. *Nat. Protoc.* 1 2320–2325. 10.1038/nprot.2006.384 17406474

[B3] Al-Sheddi E. S. Al-Oqail M. M. Saquib Q. Siddiqui M. A. Musarrat J. Al-Khedhairy A. A. (2015). Novel all trans-retinoic Acid derivatives: cytotoxicity, inhibition of cell cycle progression and induction of apoptosis in human cancer cell lines. *Molecules* 20 8181–8197. 10.3390/molecules20058181 25961160 PMC6272518

[B4] Benchabane M. Goulet C. Rivard D. Faye L. Gomord V. Michaud D. (2008). Preventing unintended proteolysis in plant protein biofactories. *Plant Biotechnol. J.* 6 633–648. 10.1111/j.1467-7652.2008.00344.x PMC7159130 18452504

[B5] Boehm R. (2007). Bioproduction of therapeutic proteins in the 21st century and the role of plants and plant cells as production platforms. *Ann. N. Y. Acad. Sci.* 1102 121–134. 10.1196/annals.1408.009 17470916 PMC7168112

[B6] Boehrer S. Chow K. U. Puccetti E. Ruthardt M. Godzisard S. Krapohl A. (2001). Deregulated expression of prostate apoptosis response gene-4 in less differentiated lymphocytes and inverse expressional patterns of par-4 and bcl-2 in acute lymphocytic leukemia. *Hematol. J.* 2 103–107. 10.1038/sj/thj/6200089 11424002

[B7] Bradford M. M. (1976). A rapid and sensitive method for the quantitation of microgram quantities of protein utilizing the principle of protein-dye binding. *Anal. Biochem.* 72 248–254. 10.1016/0003-2697(76)90527-3 942051

[B8] Burikhanov R. Zhao Y. Goswami A. Qiu S. Schwarze S. R. Rangnekar V. M. (2009). The tumor suppressor Par-4 activates an extrinsic pathway for apoptosis. *Cell* 138 377–388. 10.1016/j.cell.2009.05.022 19632185 PMC2774252

[B9] Chakraborty M. Qiu S. G. Vasudevan K. M. Rangnekar V. M. (2001). Par-4 drives trafficking and activation of Fas and Fasl to induce prostate cancer cell apoptosis and tumor regression. *Cancer Res.* 61 7255–7263. 11585763

[B10] Chang C. J. Ghosh P. K. Hu Y. F. Brueggemeier R. W. Lin Y. C. (1993). Antiproliferative and antimetastatic effects of gossypol on Dunning prostate cell-bearing Copenhagen rats. *Res. Commun. Chem. Pathol. Pharmacol.* 79 293–312. 8480076

[B11] Conley A. J. Mohib K. Jevnikar A. M. Brandle J. E. (2009). Plant recombinant erythropoietin attenuates inflammatory kidney cell injury. *Plant Biotechnol. J.* 7 183–199. 10.1111/j.1467-7652.2008.00389.x 19055608

[B12] Cook J. Krishnan S. Ananth S. Sells S. F. Shi Y. Walther M. M. (1999). Decreased expression of the pro-apoptotic protein Par-4 in renal cell carcinoma. *Oncogene* 18 1205–1208. 10.1038/sj.onc.1202416 10022126

[B13] Cooperberg M. Broering J. Carroll P. (2009). Risk assessment for prostate cancer metastasis and mortality at the time of diagnosis. *J. Natl. Cancer Inst.* 101 878–887. 10.1093/jnci/djp122 19509351 PMC2697208

[B14] Dai R. Y. Chen S. K. Yan D. M. Chen R. Lui Y. P. Duan C. Y. (2010). PI3K/Akt promotes GRP78 accumulation and inhibits endoplasmic reticulum stress-induced apoptosis in HEK293 cells. *Folia Biol. (Praha)* 56 37–46. 10.14712/fb2010056020037 20492754

[B15] Daniell H. (2006). Production of biopharmaceuticals and vaccines in plants via the chloroplast genome. *Biotechnol. J.* 1 1071–1079. 10.1002/biot.200600145 17004305

[B16] Daniell H. Muthukumar B. Lee S. B. (2001). Marker free transgenic plants: engineering the chloroplast genome without the use of antibiotic selection. *Curr. Genet.* 39 109–116. 10.1007/s002940100185 11405095

[B17] Dey N. Maiti I. B. (1999a). Further characterization and expression analysis of mirabilis mosaic virus (MMV) full-length transcript promoter with single and double enhancer domains in transgenic plants. *Transgenics* 3 61–70.

[B18] Dey N. Maiti I. B. (1999b). Structure and promoter/leader deletion analysis of mirabilis mosaic virus (MMV) full-length transcript promoter in transgenic plants. *Plant Mol. Biol.* 40 771–782. 10.1023/A:1006285426523 10487212

[B19] Downing W. L. Galpin J. D. Clemens S. Lauzon S. M. Samuels A. L. Pidkowich M. S. (2006). Synthesis of enzymatically active human alpha-L-iduronidase in *Arabidopsis* cgl (complex glycan-deficient) seeds *Plant Biotechnol. J.* 4 169–181. 10.1111/j.1467-7652.2005.00166.x 17177794

[B20] El-Guendy N. Rangnekar V. M. (2003). Apoptosis by Par-4 in cancer and neurodegenerative diseases. *Exp. Cell Res.* 283 51–66. 10.1016/S0014-4827(02)00016-2 12565819

[B21] El-Guendy N. Zhao Y. Gurumurthy S. Burikhanov R. Rangnekar V. M. (2003). Identification of a unique core domain of par-4 sufficient for selective apoptosis induction in cancer cells. *Mol. Cell. Biol.* 23 5516–5525. 10.1128/MCB.23.16.5516-5525.2003 12897127 PMC166354

[B22] Ferlay J. Shin H. R. Bray F. Forman D. Mathers C. Parkin D. M. (2010). Estimates of worldwide burden of cancer in 2008: GLOBOCAN 2008. *Int. J. Cancer* 127 2893–2917. 10.1002/ijc.25516 21351269

[B23] Folta K. M. Kaufman L. S. (2006). Isolation of *Arabidopsis* nuclei and measurement of gene transcription rates using nuclear run-on assays. *Nat. Protoc.* 1 3094–3100. 10.1038/nprot.2006.471 17406505

[B24] Fox J. L. (2006). Turning plants into protein factories. *Nat. Biotechnol.* 24 1191–1193. 10.1038/nbt1006-1191 17033647

[B25] Gleba Y. Klimyuk V. Marillonnet S. (2007). Viral vectors for the expression of proteins in plants. *Curr. Opin. Biotechnol.* 18 134–141. 10.1016/j.copbio.2007.03.002 17368018

[B26] Goddijn O. J. M. Pen J. (1995). Plants as bioreactors. *Trends Biotechnol.* 13 379–387. 10.1016/S0167-7799(00)88985-4

[B27] Goldstein D. A. Thomas J. A. (2004). Biopharmaceuticals derived from genetically modified plants. *QJM* 97 705–716. 10.1093/qjmed/hch121 15496527

[B28] Goswami A. Burikhanov R. De Thonel A. Fujita N. Goswami M. Zhao Y. (2005). Binding and phosphorylation of par-4 by akt is essential for cancer cell survival. *Mol. Cell* 20 33–44. 10.1016/j.molcel.2005.08.016 16209943

[B29] Gurumurthy S. Rangnekar V. M. (2004). Par-4 inducible apoptosis in prostate cancer cells. *J. Cell. Biochem.* 91 504–512. 10.1002/jcb.20000 14755681

[B30] Hebbar N. Wang C. Rangnekar V. M. (2012). Mechanisms of apoptosis by the tumor suppressor Par-4. *J. Cell. Physiol.* 227 3715–3721. 10.1002/jcp.24098 22552839 PMC3414654

[B31] Hwang C. Sinskey A. J. Lodish H. F. (1992). Oxidized redox state of glutathione in the endoplasmic reticulum. *Science* 257 1496–1502. 10.1126/science.1523409 1523409

[B32] Imberg-Kazdan K. Ha S. Greenfield A. Poultney C. S. Bonneau R. Logan S. K. (2013). A genome-wide RNA interference screen identifies new regulators of androgen receptor function in prostate cancer cells. *Genome Res.* 23 581–591. 10.1101/gr.144774.112 23403032 PMC3613576

[B33] Jain S. Suklabaidya S. Das B. Raghav S. K. Batra S. K. Senapati S. (2015). TLR4 activation by lipopolysaccharide confers survival advantage to growth factor deprived prostate cancer cells. *Prostate* 75 1020–1033. 10.1002/pros.22983 25833062

[B34] Jiang J. Sugimoto Y. Liu S. Chang H. L. Park K. Y. Kulp S. K. (2004). The inhibitory effects of gossypol on human prostate cancer cells-PC3 are associated with transforming growth factor beta1 (TGFbeta1) signal transduction pathway. *Anticancer Res.* 24 91–100. 15015581

[B35] Johnstone R. W. See R. H. Sells S. F. Wang J. Muthukkumar S. Englert C. (1996). A novel repressor, par-4, modulates transcription and growth suppression functions of the Wilms’ tumor suppressor WT1. *Mol. Cell. Biol.* 16 6945–6956. 8943350 10.1128/mcb.16.12.6945 PMC231698

[B36] Kapila J. Derycke R. Vanmontagu M. Angenon G. (1997). An Agrobacterium-mediated transient gene expression system for intact leaves. *Plant Sci.* 122 101–108. 10.1016/S0168-9452(96)04541-4

[B37] Kim J. S. Ku B. Woo T. G. Oh A. Y. Jung Y. S. Soh Y. M. (2015). Conversion of cell-survival activity of Akt into apoptotic death of cancer cells by two mutations on the BIM BH3 domain. *Cell Death Dis.* 6:e1804 10.1038/cddis.2015.118 PMC4650712 26136077

[B38] Kimura K. Gelmann E. P. (2000). Tumor necrosis factor-alpha and Fas activate complementary Fas-associated death domain-dependent pathways that enhance apoptosis induced by gamma-irradiation. *J. Biol. Chem.* 275 8610–8617. 10.1074/jbc.275.12.8610 10722700

[B39] Ko K. Steplewski Z. Glogowska M. Koprowski H. (2005). Inhibition of tumor growth by plant-derived mAb. *Proc. Natl. Acad. Sci. U.S.A.* 102 7026–7030. 10.1073/pnas.0502533102 15867145 PMC1100796

[B40] Kogel D. Reimertz C. Mech P. Poppe M. Fruhwald M. C. Engemann H. (2001). Dlk/ZIP kinase-induced apoptosis in human medulloblastoma cells: requirement of the mitochondrial apoptosis pathway. *Br. J. Cancer* 85 1801–1808. 10.1054/bjoc.2001.2158 11742505 PMC2363987

[B41] Kroumova A. B. Sahoo D. K. Raha S. Goodin M. Maiti I. B. Wagner G. J. (2013). Expression of an apoplast-directed, T-phylloplanin-GFP fusion gene confers resistance against *Peronospora tabacina* disease in a susceptible tobacco. *Plant Cell Rep.* 32 1771–1782. 10.1007/s00299-013-1490-6 23942845

[B42] Kroumova A. B. Shepherd R. W. Wagner G. J. (2007). Impacts of T-Phylloplanin gene knockdown and of *Helianthus* and *Datura phylloplanins* on *Peronospora tabacina* spore germination and disease potential. *Plant Physiol.* 144 1843–1851. 10.1104/pp.107.097584 17573541 PMC1949898

[B43] Kumar D. Patro S. Ranjan R. Sahoo D. K. Maiti I. B. Dey N. (2011). Development of useful recombinant promoter and its expression analysis in different plant cells using confocal laser scanning microscopy. *PLoS ONE* 6:e24627 10.1371/journal.pone.0024627 PMC3170401 21931783

[B44] Li J. Ni M. Lee B. Barron E. Hinton D. R. Lee A. S. (2008). The unfolded protein response regulator GRP78/BiP is required for endoplasmic reticulum integrity and stress-induced autophagy in mammalian cells. *Cell Death Differ.* 15 1460–1471. 10.1038/cdd.2008.81 18551133 PMC2758056

[B45] Liu Z. Fang J. Dearman J. Zhang L. Zuo J. (2014). In vivo generation of immature inner hair cells in neonatal mouse cochleae by ectopic Atoh1 expression. *PLoS ONE* 9:e89377 10.1371/journal.pone.0089377 PMC3930725 24586731

[B46] Ma J. K. Barros E. Bock R. Christou P. Dale P. J. Dix P. J. (2005a). Molecular farming for new drugs and vaccines. Current perspectives on the production of pharmaceuticals in transgenic plants. *EMBO Rep.* 6 593–599. 10.1038/sj.embor.7400470 15995674 PMC1369121

[B47] Ma J. K. Chikwamba R. Sparrow P. Fischer R. Mahoney R. Twyman R. M. (2005b). Plant-derived pharmaceuticals–the road forward. *Trends Plant Sci.* 10 580–585. 10.1016/j.tplants.2005.10.009 16290220

[B48] Ma S. Huang Y. Davis A. Yin Z. Mi Q. Menassa R. (2005c). Production of biologically active human interleukin-4 in transgenic tobacco and potato. *Plant Biotechnol. J.* 3 309–318. 10.1111/j.1467-7652.2005.00125.x 17129313

[B49] Moore A. Bonner-Weir S. Weissleder R. (2001). Noninvasive in vivo measurement of beta-cell mass in mouse model of diabetes. *Diabetes* 50 2231–2236. 10.2337/diabetes.50.10.2231 11574403

[B50] Moreno-Bueno G. Fernandez-Marcos P. J. Collado M. Tendero M. J. Rodriguez-Pinilla S. M. Garcia-Cao I. (2007). Inactivation of the candidate tumor suppressor par-4 in endometrial cancer. *Cancer Res.* 67 1927–1934. 10.1158/0008-5472.CAN-06-2687 17332319

[B51] Patro S. Maiti S. Panda S. K. Dey N. (2015). Utilization of plant-derived recombinant human β-defensins (hBD-1 and hBD-2) for averting salmonellosis. *Transgenic Res.* 24 353–364. 10.1007/s11248-014-9847-3 25417183

[B52] Plante M. K. Arscott W. T. Folsom J. B. Tighe S. W. Dempsey R. J. Wesley U. V. (2013). Ethanol promotes cytotoxic effects of tumor necrosis factor-related apoptosis-inducing ligand through induction of reactive oxygen species in prostate cancer cells. *Prostate Cancer Prostatic Dis.* 16 16–22. 10.1038/pcan.2012.37 22986577

[B53] Ranjan R. Patro S. Pradhan B. Kumar A. Maiti I. B. Dey N. (2012). Development and functional analysis of novel genetic promoters using DNA shuﬄing, hybridization and a combination thereof. *PLoS ONE* 7:e31931 10.1371/journal.pone.0031931 PMC3303778 22431969

[B54] Remans T. Schenk P. M. Manners J. M. Grof C. P. L. Elliott A. R. (1999). A protocol for the fluorometric quantification of mGFP5-ER and sGFP(S65T) in transgenic plants. *Plant Mol. Biol. Rep.* 17 385–395. 10.1023/A:1007654318401

[B55] Sahoo D. K. Dey N. Maiti I. B. (2014a). pSiM24 is a novel versatile gene expression vector for transient assays as well as stable expression of foreign genes in plants. *PLoS ONE* 9:e98988 10.1371/journal.pone.0098988 PMC4045853 24897541

[B56] Sahoo D. K. Sarkar S. Raha S. Maiti I. B. Dey N. (2014b). Comparative analysis of synthetic DNA promoters for high-level gene expression in plants. *Planta* 240 855–875. 10.1007/s00425-014-2135-x 25092118

[B57] Sahoo D. K. Ranjan R. Kumar D. Kumar A. Sahoo B. S. Raha S. (2009). An alternative method of promoter assessment by confocal laser scanning microscopy. *J. Virol. Methods* 161 114–121. 10.1016/j.jviromet.2009.06.011 19540268

[B58] Sahoo D. K. Roy A. Bhanja S. Chainy G. B. N. (2008). Hypothyroidism impairs antioxidant defence system and testicular physiology during development and maturation. *Gen. Comp. Endocrinol.* 156 63–70. 10.1016/j.ygcen.2007.11.007 18093587

[B59] Sambrook J. Fritsch E. F. Maniatis T. (1989). *Molecular Cloning: A Laboratory Manual.* Cold Spring Harbor, NY: Cold Spring Harbor Laboratory Press.

[B60] Schillberg S. Zimmermann S. Voss A. Fischer R. (1999). Apoplastic and cytosolic expression of full-size antibodies and antibody fragments in *Nicotiana tabacum*. *Transgenic Res.* 8 255–263. 10.1023/A:1008937011213 10621973

[B61] Sells S. F. Wood D. P. Jr. Joshi-Barve S. S. Muthukumar S. Jacob R. J. Crist S. A. (1994). Commonality of the gene programs induced by effectors of apoptosis in androgen-dependent and -independent prostate cells. *Cell Growth Differ.* 5 457–466. 8043520

[B62] Shukla N. Hebbar N. K. Rangnekar V. M. (2013). Role of Par-4 in Prostate Cancer. *Prostate Cancer Prostatic Dis.* 16 481–495. 10.1007/978-1-4614-6828-8_18

[B63] Siegel R. Ma J. Zou Z. Jemal A. (2014). Cancer statistics, 2014. *CA Cancer J. Clin.* 64 9–29. 10.3322/caac.21208 24399786

[B64] Tremblay R. Feng M. Menassa R. Huner N. P. Jevnikar A. M. Ma S. (2011). High-yield expression of recombinant soybean agglutinin in plants using transient and stable systems. *Transgenic Res.* 20 345–356. 10.1007/s11248-010-9419-0 20559869 PMC7477883

[B65] Vazquez F. Gonzalez E. A. Garabal J. I. Valderrama S. Blanco J. Baloda S. B. (1996). Development and evaluation of an ELISA to detect *Escherichia coli* K88 (F4) fimbrial antibody levels. *J. Med. Microbiol.* 44 453–463. 10.1099/00222615-44-6-453 8636963

[B66] Verwoerd T. C. Van Paridon P. A. Van Ooyen A. J. Van Lent J. W. Hoekema A. Pen J. (1995). Stable accumulation of *Aspergillus niger* phytase in transgenic tobacco leaves. *Plant Physiol.* 109 1199–1205. 10.1104/pp.109.4.1199 8539288 PMC157650

[B67] Vetterkind S. Boosen M. Scheidtmann K. H. Preuss U. (2005). Ectopic expression of Par-4 leads to induction of apoptosis in CNS tumor cell lines. *Int. J. Oncol.* 26 159–167. 10.3892/ijo.26.1.159 15586236

[B68] Wang D. J. Brandsma M. Yin Z. Wang A. Jevnikar A. M. Ma S. (2008). A novel platform for biologically active recombinant human interleukin-13 production. *Plant Biotechnol. J.* 6 504–515. 10.1111/j.1467-7652.2008.00337.x 18393948

[B69] Xu-Gang L. Song-Biao C. Zi-Xian L. Tuan-Jie C. Qian-Chun Z. Zhen Z. (2002). Impact of copy number on transgene expression in tobacco. *Acta Bot. Sin.* 44 120–123.

[B70] Yang Y. Li R. Qi M. (2000). In vivo analysis of plant promoters and transcription factors by agroinfiltration of tobacco leaves. *Plant J.* 22 543–551. 10.1046/j.1365-313x.2000.00760.x 10886774

[B71] Zhang G. Zhang H. Wang Q. Lal P. Carroll A. M. de la Llera-Moya M. (2014). Suppression of human prostate tumor growth by a unique prostate-specific monoclonal antibody F77 targeting a glycolipid marker. *Proc. Natl. Acad. Sci. U.S.A.* 107 732–737. 10.1073/pnas.0911397107 20080743 PMC2818935

[B72] Zhao Y. Burikhanov R. Brandon J. Qiu S. Shelton B. J. Spear B. (2011). Systemic Par-4 inhibits non-autochthonous tumor growth. *Cancer Biol. Ther.* 12 152–157. 10.4161/cbt.12.2.15734 21613819 PMC3154287

[B73] Zhao Y. Rangnekar V. M. (2008). Apoptosis and tumor resistance conferred by Par-4. *Cancer Biol. Ther.* 7 1867–1874. 10.4161/cbt.7.12.6945 18836307 PMC2683365

