# Supplementary material for: Plant-derived SAC domain of PAR-4 (Prostate Apoptosis Response 4) exhibits growth inhibitory effects in prostate cancer cells
Source: Front Plant Sci. 2015 Oct 7;6:822. doi: 10.3389/fpls.2015.00822 (PMC4595782; doi:10.3389/fpls.2015.00822)
Supplement: Supplementary file 1 [file Data_Sheet_1.DOC]

***Supplementary Material***

**Plant-derived SAC domain of PAR-4 (Prostate Apoptosis**

**Response 4) imparts anti-cancer activity**

**Shayan Sarkar^a,1^, Sumeet Jain^a,2^ , Vineeta Rai^a,1^, Dipak Kumar Sahoo^b^, Sumita Raha^c^ , Sujit Suklabaidya ^a,2^, Shantibhusan Senapati^a,2^, Vivek M. Rangnekar^d^ , Indu B. Maiti*^b^, Nrisingha Dey*^a^**

^a,1^Department of Gene Function and Regulation, Institute of Life Sciences, Department of Biotechnology, Government of India, Chandrasekharpur, Bhubaneswar, Odisha, India

^a,2^Department of Translational Research and Technology Development, Institute of Life Sciences, Department of Biotechnology, Government of India, Chandrasekharpur, Bhubaneswar, Odisha, India

^b^KTRDC, Plant Genetic Engineering Research and Services, College of Agriculture, Food and Environment, University of Kentucky, Lexington, Kentucky 40546-0236, USA

^c^Department of Radiation Oncology, Feinberg School of Medicine, Northwestern University, Ward- 13-002, 303 East Chicago Ave., Chicago IL-60611

^d^Department of Radiation Medicine, Markey Cancer Center, University of Kentucky, Lexington, KY 40536, USA

*** Correspondence:** Nrisingha Dey,

Address: Department of Gene Function and Regulation, Institute of Life Sciences, Department of Biotechnology, Government of India, Chandrasekharpur, Bhubaneswar, Odisha-751 023, India

Telephone: +91674-2300598, Fax: +91674-2300728; e-mail: nrisinghad@gmail.com

Indu Bhushan Maiti,

Address: KTRDC, College of Agriculture, University of Kentucky, Lexington, KY 40546, USA

Telephone: 1-859-257-3296, Fax: 1-859-323-1077; e-mail: [imaiti@uky.edu](mailto:imaiti@uky.edu)

**
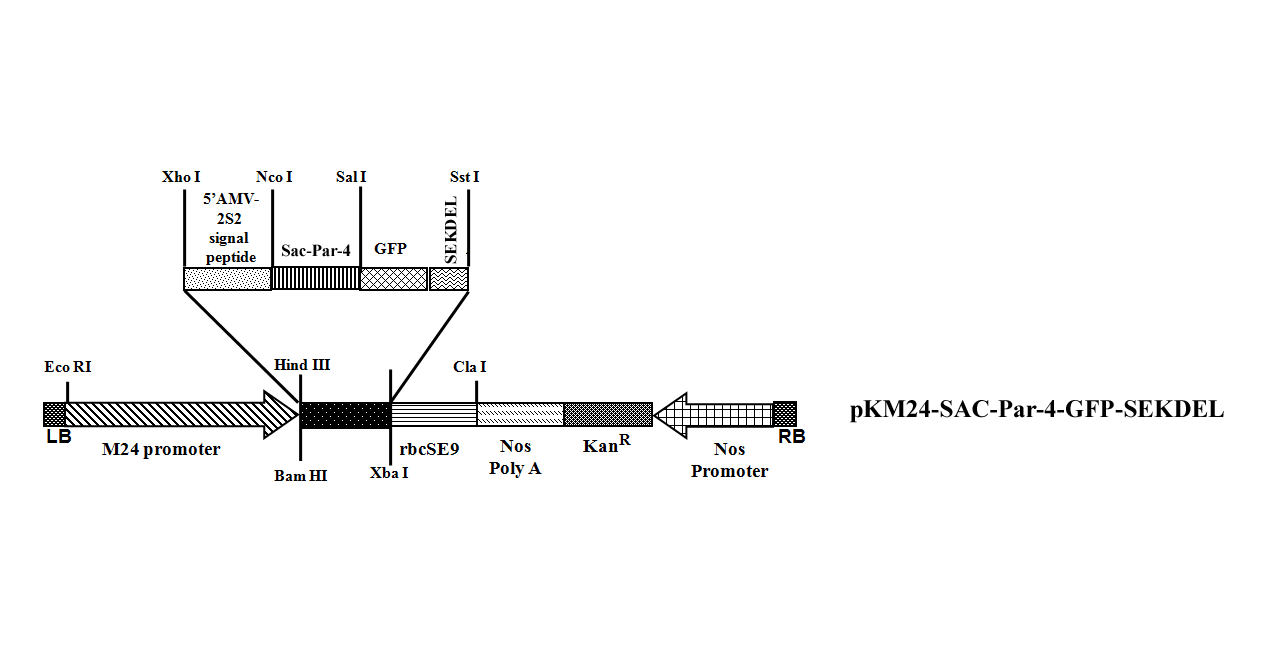
**

**Figure S1.** Schematic representation of the construct pKM24-SAC-Par-4-GFP-SEKDEL containing an additional endoplasmic reticulum (ER)-retention signal SEKDEL. LB: left T-DNA border; RB: right T-DNA border; 5′AMV: a translational enhancer sequence; aTP: the apoplast targeting sequence of the Arabidopsis 2S2 protein; M24: recombinant full-length *Mirabilis mosaic virus* promoter, Kan^R^: neomycin phosphotransferase II marker gene; rbcSE9: the 3′-terminator sequences (terminators) of the ribulose bisphosphate carboxylase small subunit and Nos PolyA: nopaline synthase genes are shown. The *EcoR*I, *Xho*I, *Nco*I, *Sal*I, *Sst*I, *Hind*III, *Bam*HI, *Xba*I and *Cla*I restriction sites used to assemble these expression vectors are shown.

**
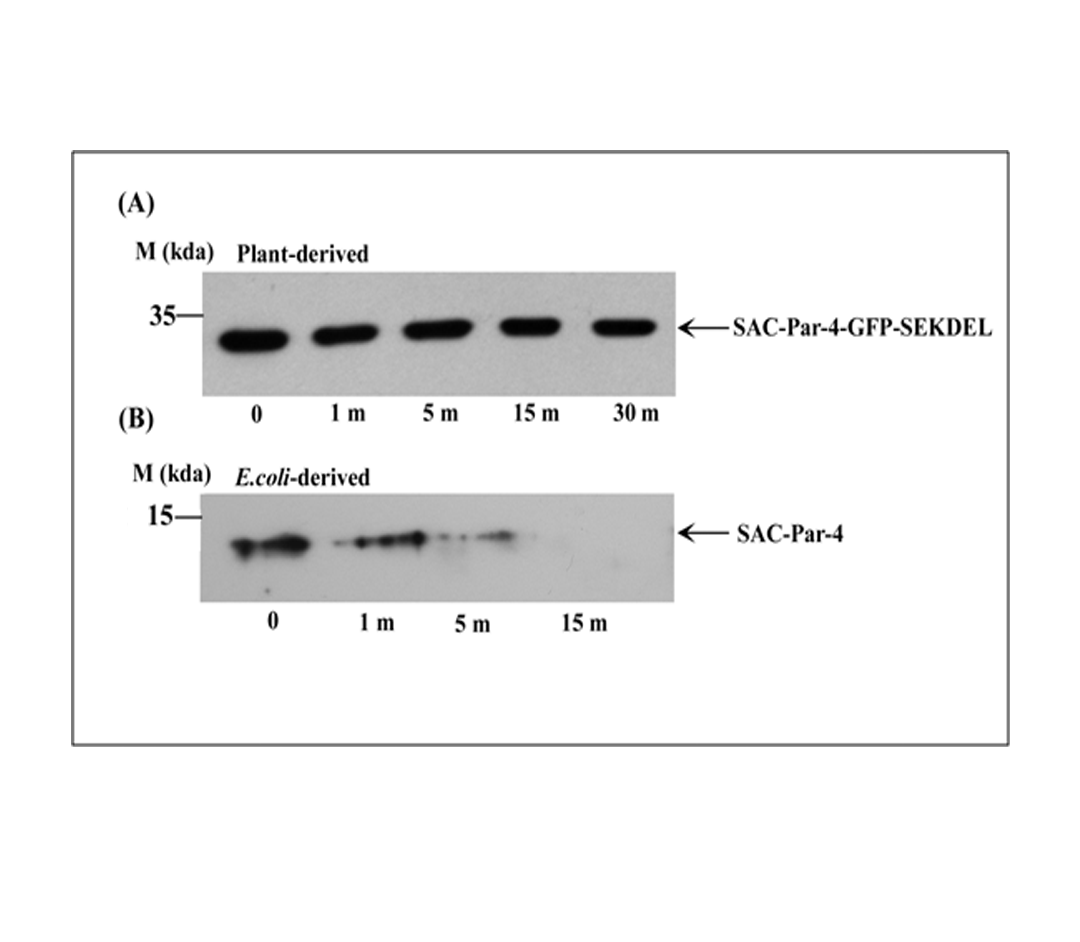
**

**Figure S2.** Proteolysis assay. Trypsin digestion of recombinant SAC-Par-4 protein for various time increments and western blot analysis using anti-Par-4 antibody of (a) transiently expressed SAC-Par-4-GFP-SEKDEL protein (b) bacterial derived SAC-Par-4. Each lane contains the starting equivalent of approximately 500 ng total protein. The position of the molecular weight marker (M) is indicated.

**
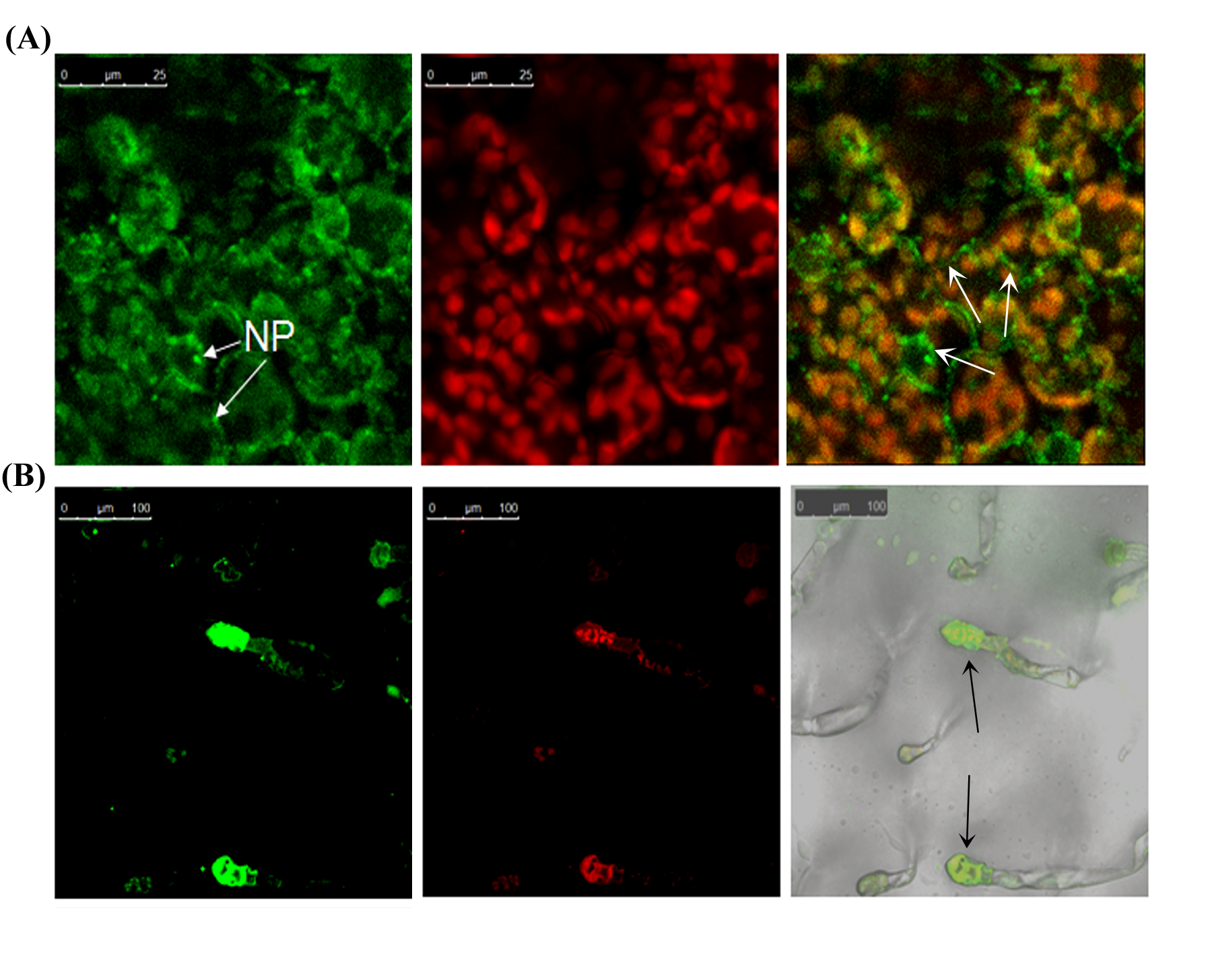
**

**Figure S3.** Confocal images of tobacco leaf epidermal cells (A) and leaf trichomes (B) of transgenic tobacco line L3 expressing pKM24-SAC-Par-4-GFP in the apoplasm. (A) Apoplastic GFP fluorescence is detected in the cytoplasmic layer at the periphery of guard cells and at nucleoplasm (NP). Superimposed images were obtained to image red fluorescence of chloroplasts and imaging of GFP. Scale bars are shown for each panel.
